# Plasticity of histone modifications around Cidea and Cidec genes with secondary bile in the amelioration of developmentally-programmed hepatic steatosis

**DOI:** 10.1038/s41598-019-52943-7

**Published:** 2019-11-19

**Authors:** Jeenat Ferdous Urmi, Hiroaki Itoh, Keiko Muramatsu-Kato, Yukiko Kohmura-Kobayashi, Natsuyo Hariya, Divyanu Jain, Naoaki Tamura, Toshiyuki Uchida, Kazunao Suzuki, Yoshihiro Ogawa, Nobuaki Shiraki, Kazuki Mochizuki, Takeo Kubota, Naohiro Kanayama

**Affiliations:** 1 Department of Obstetrics and Gynecology, Hamamatsu University School of Medicine, Hamamatsu, Japan; 2 Department of Nutrition, Faculty of Health and Nutrition, Sciences, Yamanashi Gakuin University, Yamanashi, Japan; 3 Department of Medicine and Bioregulatory Science, Graduate School of Medical Sciences, Kyushu University, Fukuoka, Japan; 4 Department of Molecular and Cellular Metabolism, Graduate School of Medical and Dental Sciences, Tokyo Medical and Dental University, Tokyo, Japan; 5 Department of Life Science and Technology, School of Life Science and Technology, Tokyo Institute of Technology, Yokohama, Japan; 6 Laboratory of Food and Nutritional Sciences, Department of Local Produce and Food Sciences, Faculty of Life and Environmental Sciences, University of Yamanashi, Yamanashi, Japan; 7 Faculty of Child Studies, Seitoku University, Matsudo, Japan

**Keywords:** Non-alcoholic fatty liver disease, Nutrition

## Abstract

We recently reported that a treatment with tauroursodeoxycholic acid (TUDCA), a secondary bile acid, improved developmentally-deteriorated hepatic steatosis in an undernourishment (UN, 40% caloric restriction) ***in utero*** mouse model after a postnatal high-fat diet (HFD). We performed a microarray analysis and focused on two genes (Cidea and Cidec) because they are enhancers of lipid droplet (LD) sizes in hepatocytes and showed the greatest up-regulation in expression by UN that were completely recovered by TUDCA, concomitant with parallel changes in LD sizes. TUDCA remodeled developmentally-induced histone modifications (dimethylation of H3K4, H3K27, or H3K36), but not DNA methylation, around the Cidea and Cidec genes in UN pups only. Changes in these histone modifications may contribute to the markedly down-regulated expression of Cidea and Cidec genes in UN pups, which was observed in the alleviation of hepatic fat deposition, even under HFD. These results provide an insight into the future of precision medicine for developmentally-programmed hepatic steatosis by targeting histone modifications.

## Introduction

Non-alcoholic fatty liver disease (NAFLD) is a hepatic manifestation of metabolic syndrome with a global prevalence of 24%, including South America and the Middle East, followed by Asia, the USA, and Europe^1–3^. Evidence to support the relationship between nutritional imbalances in the early developmental period and a predisposition for NAFLD in later life is increasing^4–9^. Sandboge *et al*. examined 1587 aged participants from the Helsinki Birth Cohort Study and showed that birth and childhood body sizes were negatively associated with NAFLD outcomes and also that individuals who had been small in early life and obese as adults were at the highest risk of developing NAFLD^6^. Faientza *et al*. reported that NAFLD was detected in 34.8% of children who were born as small for gestational age (SGA), but not in those born as appropriate for gestational age^7^. Alisi *et al*. showed that SGA was present in 38.9% of children complicated with NAFLD and in 6.7% of uncomplicated children^8^. Moreover, Bugianesi *et al*. found that a low birthweight increased the likelihood of severe steatosis in pediatric NAFLD^4^. Ours and previous studies demonstrated that maternal global nutrient restrictions primed the deterioration of hepatic steatosis in adulthood in ovine^10^, rat^11^, and mouse^12^ offspring. Collectively, the findings of human cohorts and animal studies support the concept that undernourishment (UN) *in utero* primes the risk of augmented hepatic fat deposition in later life, particularly with an obesogenic diet. However, the underlying mechanisms remain unclear. Alisi *et al*. recently demonstrated the importance of pre- and postnatal environmental monitoring for protection against NAFLD^9^; however, specific therapeutic strategies have not yet been established, particularly after the progression of NAFLD originating from nutritional imbalances during early life.

We established mouse models of UN *in utero* by maternal caloric retraction and developing the phenotypes of various non-communicable diseases^12–16^, and subsequently demonstrated that treatments with the hydrophilic secondary bile acid tauroursodeoxycholic acid (TUDCA), an endoplasmic reticulum (ER) stress alleviator, markedly ameliorated developmentally-deteriorated hepatic steatosis^12^. The TUDCA treatment was only effective for pups with UN *in utero*. A previous study reported that a treatment with TUDCA improved many acute and chronic diseases^17^; however, the underlying mechanisms have not yet been elucidated.

In the developmental origins hypothesis, epigenetic modifications, such as DNA methylation and histone modifications, are considered to play critical roles in mediating how the early life environment impacts on later health and susceptibility to non-communicable diseases^18,19^. Moreover, increasing evidence has revealed the extensive involvement of epigenetic modifications in the pathophysiology of NAFLD^20,21^.

Therefore, we hypothesize that (1) UN *in utero* may epigenetically program the expression of some genes, by DNA methylation and/or histone modifications, in the process of the deterioration of hepatic steatosis in offspring in a mouse model of UN *in utero* under an obesogenic diet, and (2) a treatment with TUDCA remodels these epigenetic modifications, concomitant with the amelioration of advanced hepatic steatosis originating from UN *in utero*, as an example of epigenetic plasticity in the developmental programming of specific genes.

To prove these hypotheses, we investigated genetic profiles in the liver using a microarray analysis and epigenetic regulation by methyl-binding domain (MBD) protein sequencing and chromatin immunoprecipitation (ChIP) assays.

## Results

### Deterioration of hepatic steatosis by UN *in utero* under the obesogenic diet

**Figure 1:**
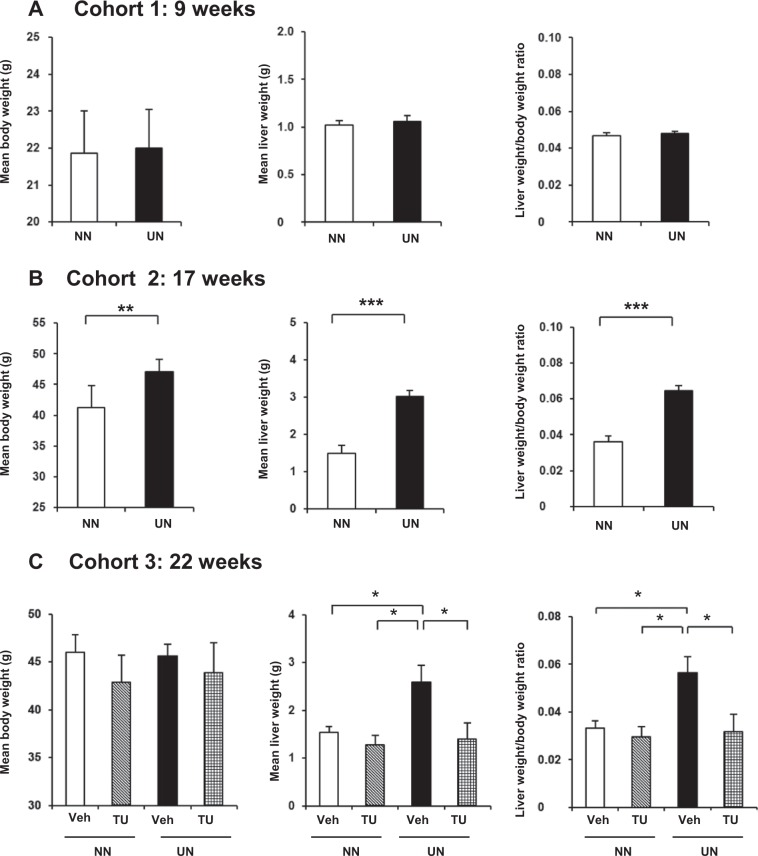
Liver and body weight changes by HFD simulating hepatic steatosis. Data are expressed as means and error bars indicate standard deviations (SD) in cohort 1 (**A**), cohort 2 (**B**), and cohort 3 (**C**). Significant differences were observed using the Student’s *t*-test (**A,B**) or Steel-Dwass test (**C**) (p* < 0.05, p** < 0.01, p*** < 0.001).

**Figure 2:**
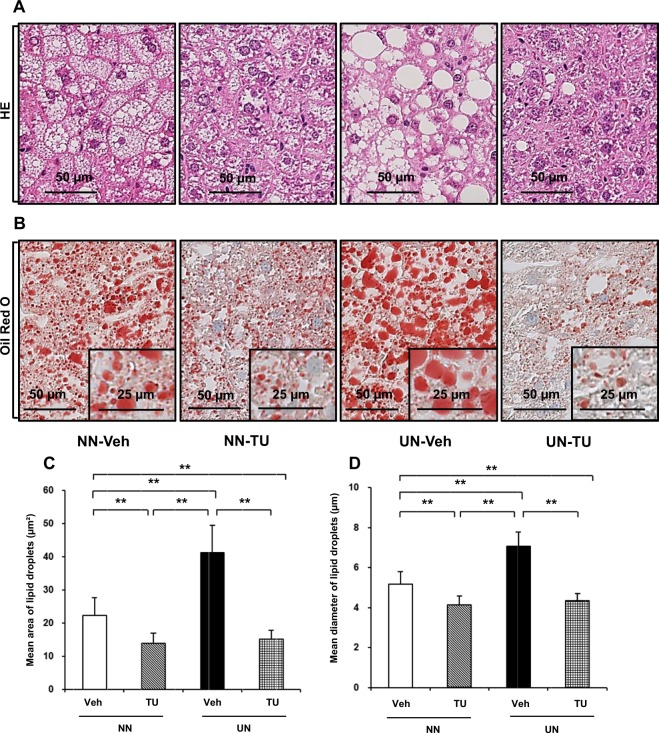
Enlargements in LD are consistent with the deterioration of hepatic steatosis and restoration with TUDCA in cohort 3. Under ×40 magnification; (**A**) HE stain, (**B**) Oil Red O stain (inset with ×80 magnification), (**C**) mean area of LD, (**D**) mean diameter of LD with error bars indicating SD. Significance is stated as p** < 0.01 calculated by the Steel-Dwass test.

UN induced a significant deterioration in hepatic steatosis after HFD (cohorts 2 and 3), but not before HFD (cohort 1) (Fig. 1 and Supplementary Fig. S1A–K). The administration of TUDCA significantly ameliorated hepatic steatosis for UN *in utero* only (Figs 1C, 2A). Figure 2 shows increases in LD sizes along with the deterioration after HFD, and the restoration of their size after the administration of TUDCA. These results were consistent with our previous findings^12^.

### Changes in gene expression profiles with or without the TUDCA treatment

**Figure 3:**
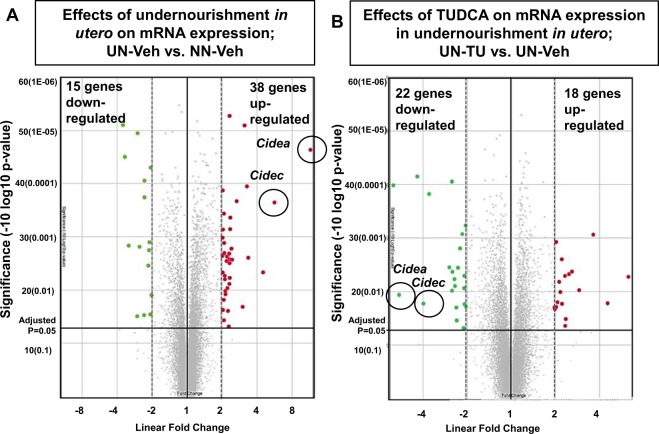
Genetic profiling of developmentally-programmed hepatic steatosis by a microarray analysis; differentially expressed mRNA in UN *in utero* and alterations by TUDCA. A Volcano plot of 34,472 genes expressed in all three cohorts. Red and green dots were up- and down-regulated genes, respectively, in cohort 3 (Linear fold change of ≤−2 and ≥2 and ANOVA P < 0.05). Cidea and Cidec genes are encircled on the plot, and were up-regulated in UN-Veh and then down-regulated with the oral gavage of TUDCA.

To examine the entire genetic profile, we performed a microarray analysis of all 3 cohorts (at the end of 9, 17, and 22 weeks). A total of 34,472 genes were expressed among all groups. The microarray analysis of cohorts 1 and 2, before and after starting HFD, revealed that 8 and 82 genes were differentially expressed, respectively (Linear fold change ≤−2 or ≥2 fold, ANOVA p < 0.05, UN-Veh vs. NN-Veh) (Supplementary Table S1). Similarly, in cohort 3 at 22 weeks, we examined 53 genes (Linear fold change ≤−2 or ≥2 fold, ANOVA p < 0.05, UN-Veh vs. NN-Veh) shown with a Volcano plot (Fig. 3A). The TUDCA treatment also altered the expression of genes in NN-TU (69 genes; Linear fold change ≤−2 or ≥2 fold, ANOVA p < 0.05) and UN-TU (40 genes; Linear fold change ≤−2 or ≥2 fold, ANOVA p < 0.05) (Fig. 3B). In this cross-sectional assessment, we stratified these genes to select 15 candidate genes in cohort 3 that were significantly up- or down-regulated in UN *in utero* and restored by TUDCA, as listed in Supplementary Tables S1 and S2. We also performed a longitudinal assessment of microarray data between cohorts 1 and 2. We studied 133 genes, listed in Supplementary Table S3, which showed significant changes in comparisons between UN before HFD (cohort 1) and UN after HFD (cohort 2). We then contrasted them with those unaltered between NN before HFD (cohort 1) and NN after HFD (cohort 2). Therefore, in this present study, we enlisted 9 genes of interest (GOI) in cross-sectional (cohort 3) and longitudinal analyses (between cohorts 1 and 2) (Table 1). We submitted our microarray data to the GEO repository, which is approved under the accession number GSE123733.

**Table 1:** List of genes of interest (GOI).

Gene Symbol	Linear Fold Changes (UN in cohort 2 vs cohort 1)	ANOVA P values (UN in cohort 2 vs cohort 1)	Linear Fold Changes (NN in cohort 2 vs cohort 1)	ANOVA P values (NN in cohort 2 vs cohort 1)	Linear Fold Changes (UN in cohort 3 vs cohort 2)	ANOVA P values (UN in cohort 3 vs cohort 2)	Linear Fold Changes (In cohort 3; UN-Veh vs. NN-Veh)	ANOVA P values (In cohort 3; UN-Veh vs. NN-Veh)	Linear Fold Changes (In cohort 3; UN-TU vs. UN-Veh)	ANOVA P values (In cohort 3; UN-TU vs. UN-Veh)
Rgs16	9.67	0.000	5.71	0.44	1.55	0.218	4.51	0.004	−4.24	0.097
Cidec	8.77	0.001	2.87	0.07	1.64	0.162	5.6	0.000	−3.99	0.017
Cidea	5.68	0.010	1.62	0.26	3.04	0.009	11.49	0.000	−5.82	0.011
Themis	5.09	0.003	2.41	0.00	−1.24	0.453	2.31	0.003	−3.66	0.000
Ifi27l2b	4.06	0.000	2.49	0.00	1.01	0.885	2.09	0.037	−2.44	0.006
Cyp17a1	4.04	0.018	7.63	0.00	1.57	0.207	−2.58	0.002	2.89	0.009
Sprr1a	3.09	0.022	1.73	0.17	1.04	0.806	2.4	0.002	−2.38	0.020
Ly6d	2.64	0.001	1.3	0.12	1.12	0.817	2.98	0.021	−2.42	0.008
Orm3	2.53	0.000	1.1	0.50	1.44	0.079	3.35	0.002	−4.39	0.000

Microarray analysis of cohorts 1, 2, and 3: 9 Candidate genes were selected after a thorough assessment of microarray data using longitudinal and cross-sectional analyses. In the cross-sectional analysis of cohort 3, 15 genes were significantly altered by UN *in utero* (≤−2 or ≥2 linear fold change and ANOVA P value < 0.05) and significantly restored by the TUDCA treatment. The longitudinal analysis identified 133 genes that were altered by UN *in utero* after HFD (cohort 1 vs cohort 2, ≤−2 or ≥2 linear fold change and ANOVA P value < 0.05).

**Figure 4:**
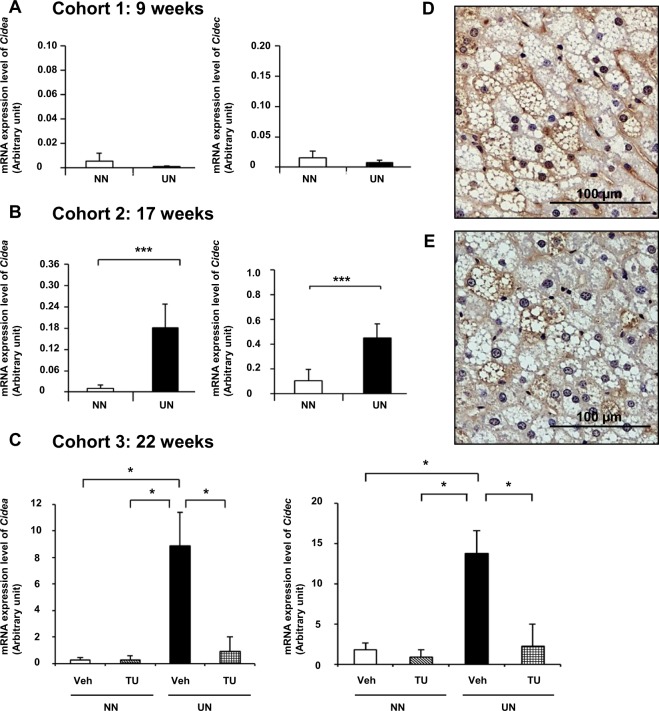
Gene expression and immunohistochemistry of Cidea and Cidec. Quantitative RT-PCR of Cidea and Cidec in cohort 1 (**A**), cohort 2 (**B**), and cohort 3 (**C**). Immunohistochemistry of Cidea (**D**) and Cidec (**E**). Significance was observed by the Student’s *t*-test (**A,B**) or Steel-Dwass test (**C**) (p* < 0.05 and p*** < 0.001).

Cell Death-Inducing DNA Fragmentation Factor-Like Effectors A (Cidea) and C (Cidec) were included in both of the analyses. This particular pattern of expression of these two genes was observed between 9 and 22 weeks (Supplementary Table S4). Relative quantitative RT-PCR showed that the mRNA expression of the Cidea and Cidec genes in cohorts 1, 2, and 3 was consistent with microarray data (Fig. 4A–C). Cidea and Cidec are known to increase LD sizes, thereby contributing to lipid storage. Changes in LD sizes in cohorts 2 and 3 were consistent with those in the gene expression of Cidea and Cidec (Fig. 2B–D and Supplementary Fig. S1L-O). Moreover, the presence of Cidea and Cidec proteins was widely observed in livers with steatosis by immunohistochemistry (Fig. 4D,E).

Therefore, we performed an epigenetic assessment of Cidea and Cidec because of their high fold changes and potential ability to regulate hepatic fat deposition.

### DNA methylation alterations in Cidea and Cidec genes

Large methylation variations were observed among the four groups and a list of methylated genes was shown in Supplementary Table S5. We examined the entire genome lengths of the Cidea (chr18:67320000–67410000) and Cidec (chr6:113415000–113450000) genes, including up- and downstream regions. No significant differences were observed in methylation peaks among the four groups before and after the TUDCA treatment (Supplementary Fig. S2).

### Effects of UN *in utero* on ChIP assay results around Cidea

**Figure 5:**
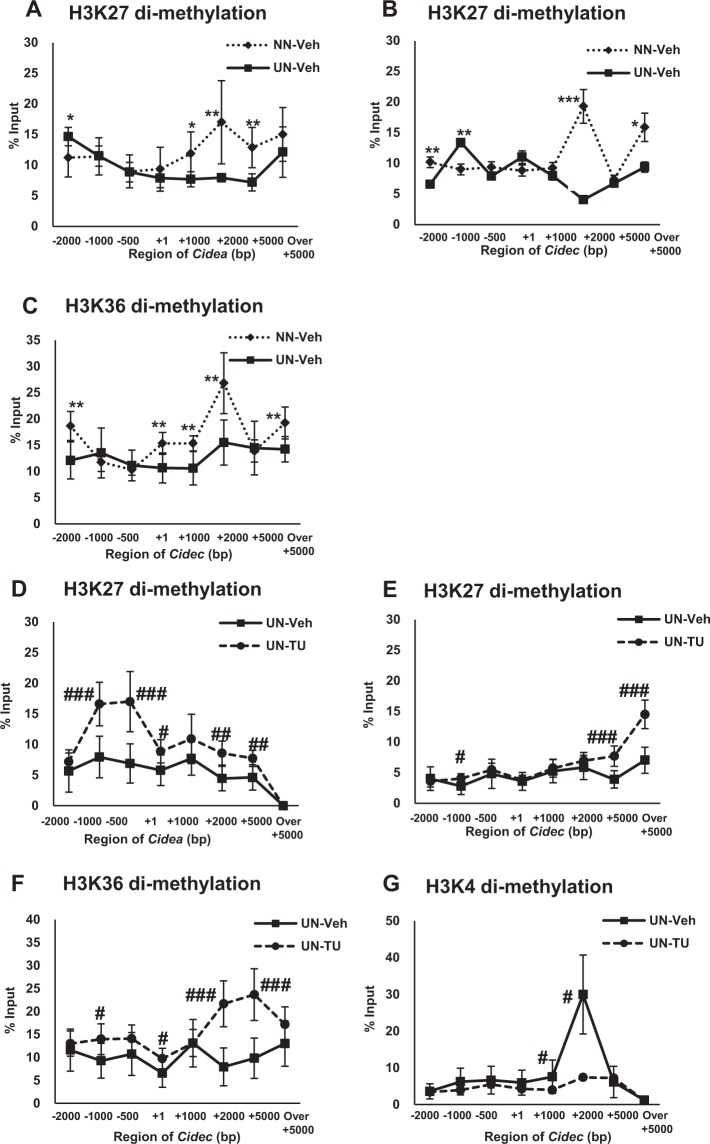
Histone modifications by the ChIP assay. Effects of UN *in utero* on the dimethylation of H3K27 around Cidea (**A**) and dimethylation of H3K27 (**B**) and H3K36 (**C**) around Cidec. Effects of TUDCA and UN *in utero* on H3K27 around Cidea (**D**) and the dimethylation of H3K27 (**E**), H3K36 (**F**), and H3K4 (**G**) around Cidec. Mean values were expressed with SD as error bars and significance (*or ^#^p < 0.05, **or ^##^p < 0.01, ***or ^###^p < 0.001) was calculated with the Student’s *t*-test or Mann-Whitney U test as appropriate. The X-axis shows the region of gene length and the y-axis is % input.

In cohort 3, we performed a ChIP assay to examine the acetylation and methylation levels of H3 and H4 around the Cidea and Cidec genes (Fig. 5 and Supplementary Fig. S3–7). Around the Cidea gene, a modification known to inhibit gene expression^22^, i.e. the level of dimethylation of H3K27, was significantly suppressed at the 3′ end (Fig. 5A). This result was consistent with the elevated mRNA expression levels of Cidea in the microarray assay as well as qPCR for UN *in utero* (Fig. 4C).

### Effects of UN *in utero* on ChIP assay results around Cidec

UN *in utero* caused changes in various histone modifications around Cidec, contributing to strong genetic expression. Around the Cidec gene, the levels of gene suppressor modifications, the dimethylation of H3K27 and H3K36^23^, were lower near the 3′ end (Fig. 5B,C). These results were consistent with the augmented mRNA expression levels of Cidec in the microarray assay as well as qPCR for UN *in utero* (Fig. 4C).

### Effects of the TUDCA treatment on ChIP assay results around Cidea in UN offspring

TUDCA enhanced the suppressor modification H3K27 dimethylation in the entire length, enriched at the 3′ end, to inhibit the mRNA expression of Cidea, as shown by the microarray analysis and qPCR (Fig. 5D). This was consistent with the decrease in the mRNA expression levels of Cidea in the microarray assay as well as qPCR for UN *in utero* with the TUDCA treatment (Fig. 4C).

### Effects of the TUDCA treatment on ChIP assay results around Cidec in UN offspring

The H3K27 and H3K36 dimethylation modifications around Cidec were markedly enhanced by TUDCA in UN *in utero*, and were also observed near the 3′ end (Fig. 5E,F), restoring changes in mRNA expression. Moreover, lower levels of activator modifications, such as H3K4 dimethylation, were also prominent in TUDCA-treated UN *in utero* pups (Fig. 5G) at the 3′ end, which is suggestive of remodeling. These results were consistent with the low mRNA expression levels of Cidec in the microarray assay as well as qPCR for UN *in utero* with the TUDCA treatment (Fig. 4C).

### Effects of the TUDCA treatment on ChIP assay results around Cidea and Cidec in NN offspring

The TUDCA treatment exerted suppressive (H3K4 and H3K36 dimethylation) and enhancing (H3K27 dimethylation) effects on the gene expression of Cidea and Cidec in NN offspring (Supplementary Figs S6, S7). We currently cannot provide an explanation for the contribution of histone modifications to gene expression in NN offspring because the TUDCA treatment induced changes that were not consistent for the construction of histones (Fig. 4C).

## Discussion

In the present study, we performed a microarray analysis of developmentally-deteriorated hepatic steatosis and its amelioration by TUDCA^12^. Cross-sectional and longitudinal analyses of microarray data were performed and 9 GOI (Table 1) were commonly selected.

Among the 9 GOI examined, the Cidea and Cidec genes were markedly up-regulated and reported to be closely related to the pathophysiology of metabolic disorders, such as diabetes, obesity, and, most importantly, liver steatosis in humans as well as in animals^24,25^. The gene enrichment analysis with the functional annotation of 9 GOI showed that Cidea and Cidec had the greatest involvement in the function of apoptotic pathways and also LD and lipid particle pathways (Supplementary Fig. S8). Cidea and Cidec have been shown to increase LD sizes, thereby augmenting fat deposition in hepatocytes^24,26^, and their strong expression by UN and normalization by the TUDCA treatment (Fig. 4C) explained the deterioration induced by UN and amelioration by TUDCA in hepatic steatosis in our mouse model. Immunohistochemistry revealed the wide distribution of the Cidea and Cidec proteins in LD of various sizes in the mouse fatty liver (Fig. 4D,E). Oil Red O staining showed the significant enlargement of LD with UN *in utero*, and this was markedly reduced by the TUDCA treatment (Fig. 2B–D). The latest definition of hepatic steatosis involves the excessive accumulation of LD, which was previously hypothesized to be triggered by Cide proteins, including Cidea and Cidec^26–28^. Since 7 other GOIs appeared to be of less biological relevance to the pathophysiology of hepatic steatosis than Cidea and Cidec (Supplementary Table S6), we focused on Cidea and Cidec.

The emerging concept of epigenetics has emphasized the impact of gestational nutrition over the regulation of gene expression in the development of non-communicable diseases^18,29^. The critical involvement of epigenetic modifications has been noted in the mechanistic background of the developmental origins hypothesis^18,19^ as well as the pathophysiology of NAFLD^20,21,30^. Therefore, we investigated whether epigenetic modifications around the Cidea and/or Cidec genes are examples of epigenetic plasticity in the UN-induced deterioration as well as TUDCA-induced amelioration of hepatic steatosis in this animal model.

We then investigated significant differentially methylated sites using overlapping peaks by DNA MBD sequencing. Neither maternal caloric restriction nor the TUDCA treatment had any effect on DNA methylation around entire Cidea and Cidec genes, including up- and downstream regions (Supplementary Fig. S2). Therefore, further studies are warranted to evaluate the role of chromatin modifications.

Environmental factors, such as diet, are known to induce gene expression by histone modifications that include acetylation and methylation at lysine (K) and arginine within the histone tail^18,29,31^. Therefore, we investigated the mono- and dimethylation of H3K9, H3K27, and H3K36, dimethylation of H3K4, trimethylation of H3K9, H3K27, and H4K20, and acetylation of H3K9 and H4 (Fig. 5 and Supplementary Figs S3–10). Among 12 modifications, significant changes were observed in the dimethylation of H3K27, H3K36, and H3K4 (Fig. 5).

UN *in utero* suppressed H3K27 dimethylation in the gene body of Cidea, thereby contributing to the up-regulation of mRNA (Fig. 5A). In contrast, the dimethylation of both H3K27 and H3K36 (Fig. 5B,C) was suppressed in the gene body of Cidec, and positively correlated with up-regulated mRNA expression in microarrays and qPCR. Histone modifications around the gene body region have been reported to regulate gene expression via transcription elongation^32–35^. The present results showed a greater likelihood of the suppression of inhibitory effects on transcriptional elongation around the gene body^34,35^.

TUDCA is a secondary bile acid that acts on the H3 protein under non-enzymatic conditions to induce post-translational modifications in humans^31^. In the present study, the ChIP assay showed a clear relationship between the TUDCA treatment and its impact on the regulation of histone modifications in offspring. For example, TUDCA augmented the dimethylation of H3K27^22^ around Cidea, which induced an inhibitory effect on transcriptional elongation through upstream to the gene body (Fig. 5D). Similarly, TUDCA accelerated the inhibitory effects of the dimethylation of H3K27 and H3K36^34,35^ around Cidec on transcriptional elongation in the gene body (Fig. 5E,F). These modifications resulted in a marked decrease in the mRNA expression of Cidea and Cidec, respectively. Gene loci remodeled by TUDCA were not identical to those by UN (Fig. 5). Moreover, TUDCA induced new histone modifications, such as H3K4 dimethylation around the Cidec gene (Fig. 5G), which inhibited stimulatory effects on transcriptional elongation in the Cidec gene body^33^, leading to the suppression of Cidec gene expression. Therefore, we speculate that TUDCA may not restore, but remodel histone modifications around Cidea and Cidec. Furthermore, TUDCA may restore gene expression by repressing mRNA transcription elongation concomitant with the amelioration of hepatic steatosis. In contrast, we did not detect significant alterations in H3K9 or H3K27 trimethylation and only slight changes in H4K20 by UN *in utero*, without any effect of TUDCA (Supplementary Fig. S9A–F), even though these have been regarded as important repressors of mRNA transcription^22,36–38^.

Recent studies demonstrated that H3K27 methylation may regulate transcription repression independent of DNA methylation by environmental influences^39^, which supports the direct involvement of H3K27 methylation around Cidea and Cidec in their gene expression without significant changes in DNA methylation.

The expression of the Cidea and Cidec genes was dormant from the early period up to 9 weeks in all groups. However, HFD between weeks 17 and 22 gradually induced the strong expression of the Cidea and Cidec genes (Fig. 4) and encoded their expression prominently in pups with UN *in utero* collateral to the deterioration of hepatic steatosis, as evidenced by lipid profiles (Supplementary Fig. S1).

Interesting results were obtained for histone modifications in cohorts 1 and 2 (Supplementary Fig. S10). In Cidea, although H3K27 dimethylation was not affected by UN *in utero* at 9 weeks (cohort 1), HFD led to partial inhibition, which persisted long-term through to 22 weeks (Supplementary Fig. S10A,B). H3K36 dimethylation was transiently inhibited after HFD at 17 weeks; however, no significant changes were observed at 9 or 22 weeks (Supplementary Fig. S10C,D). This result suggests modifications involved in the plasticity of histone remodeling. H3K27 dimethylation showed the similar persistency of suppression in Cidec (Supplementary Fig. S10E,F). However, H3K36 dimethylation remained unchanged even after HFD, and long-term environmental exposure led to the suppression of this modification (Supplementary Fig. S10G,H). H3K4 dimethylation in Cidec showed minor suppression at 9 weeks, but no effect between 17 and 22 weeks (Supplementary Fig. S10K,L), suggesting that the TUDCA treatment specifically affects H3K4 dimethylation in Cidec in UN offspring (Fig. 5G). This distinctive pattern of expression indicates that these histone modifications are preprogrammed via an unidentified mechanism by the maternal nutritional state *in utero* and HFD triggered subsequent effects in a stepwise manner.

In the present study, we did not investigate the direct mechanism by which the TUDCA treatment changed histone modifications. However, the ChIP assay on the Histone 3.3 subunit, an important recruiter of methyl-transferase^40^, did not show any alterations around the Cidea and Cidec genes after the administration of TUDCA (Supplementary Fig. S9G,H). These results suggest that this subtype deposition does not play major roles in TUDCA-induced alterations in H3 methylation; however, we cannot deny that TUDCA may play a role after the recruitment of methyl-transferase around these genes.

TUDCA is a well-established ER stress alleviator and we previously revealed the significant suppression of the ER stress response in the liver by a treatment with TUDCA^12^. Since ER stress has been reported to modulate the activities of H3K4 methyltransferases in various organs^41–43^, we speculate that reductions in ER stress by TUDCA may induce histone modifications in our study model through unidentified mechanisms. Cidea and Cidec are present in the ER membrane^24^ and ER stress has been shown to regulate the formation of LD with metabolic disruption^44^. We intend to investigate the involvement of ER stress in the plasticity of histone modifications around the Cidea and Cidec genes following a treatment with TUDCA.

The effects of TUDCA on histone modifications around Cidec were more prominent than those around Cidea, suggesting that an unidentified mechanism is involved in the developmental programming of Cide proteins and their phenotypic expression. TUDCA-inducing histone re-modifications were observed in accordance with the normalization of Cidea and Cidec gene expression levels as well as the marked amelioration of hepatic steatosis, even under HFD in UN, but not NN offspring (Supplementary Figs S6–7).

There were some limitations in the present study. We used the cut-off value of a two-fold change and ANOVA P values in the microarray analysis, in accordance with our previous studies^45,46^; however, we cannot fully deny the possible contribution of genes with less than two-fold changes. We were unable to assess quantitative changes in Cidea and Cidec protein expression due to the technical limitations of the antibodies available. Furthermore, we also did not examine any knockdown or knock-in *in vivo* or *in vitro* models to prove whether Cidea and/or Cidec are the indispensable contributors in this specified animal model. However, a recent finding showed that the partial silencing of the expression of *Cide* genes improved fatty liver in a mouse model^47^. In the study of histone modifications around Cidea and Cidec, we performed multiple qPCRs after immunoprecipitation, and due to our technical limitations, we were unable to conduct next-generation sequencing. Therefore, the positions of specific modifications were not mapped.

In conclusion, using the present experimental model, we demonstrated plasticity in developmentally-programmed histone modifications around the Cidea and Cidec genes during the amelioration of hepatic steatosis by a treatment with TUDCA. The present study has provided insights into the therapeutic targets of histone modifications for the future of precision medicine for developmentally-programmed hepatic steatosis.

## Materials and Methods

### Animal model

**Figure 6:**
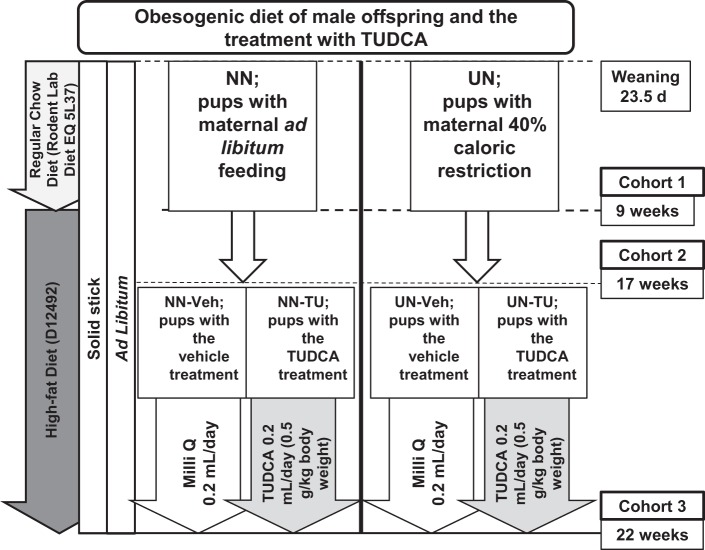
Schematic presentation of the undernourishment *in utero* mouse model: male offspring under an obesogenic diet. Cohort 1: After weaning, NN pups from *ad libitum* NN dams and UN pups from calorie-restricted UN dams were fed a regular chow diet up to 9 weeks. Cohort 2: High-fat diet (HFD) between 9 and 17 weeks. Cohort 3: Pups were randomly subdivided into vehicle groups (NN-Veh and UN-Veh) and TUDCA-treated groups (NN-TU and UN-TU) still under HFD up to 22 weeks.

The mouse model of UN *in utero* has been well established in our previous studies^12–16^. Different time points of 3 independent cohorts were described in Fig. 6, as previously reported^12^. Pregnant C57Bl/6NCr mice at 7.5 days post-conception (dpc) were purchased from Japan SLC, Inc. (Hamamatsu, Japan) and fed a regular chow diet (formula number D06121301, Research Diets Inc., New Brunswick, NJ) under a 12-h light/dark cycle. On 11.5 dpc, dams were randomly divided into an *ad libitum* or normal nourished group (group NN dams; n = 20) and a caloric restriction or undernourished group (group UN dams; n = 20). Group NN dams were fed powdered regular chow *ad libitum* and group UN dams were fed a 40% calorie reduction i.e. 60% of the average daily intake of group NN dams, from 11.5 dpc to the day before the delivery of pups (18.5 dpc). We used male offspring only and adjusted the number to 8 pups per litter and cross-fostered (on 1.5 days of age) them with NN dams (up to 23.5 days of age)^12^. Pups were then fed the regular chow diet for 1 week (up to 9 weeks) followed by a high-fat diet (HFD) containing 60% lipids (formula number D12492, Research Diets Inc.) up to 22 weeks in order to mimic an obesogenic diet, as previously described^12^. We initiated different experimental procedures with 3 cohorts as follows: cohort 1 (at 9 weeks; without HFD), cohort 2 (at 17 weeks; with HFD), and cohort 3 (at 22 weeks; HFD with or without the TUDCA treatment)^12^. In cohort 3, TUDCA (Merck Japan Ltd., Tokyo, Japan) was orally administered by gastric lavage at 0.5 g/kg body weight per day while pups were on HFD, according to our previous study^12^ (Fig. 6). All animal procedures were approved by the Institutional Animal Research Committee of Hamamatsu University School of Medicine (H20–014) and conducted in accordance with the ARRIVE guidelines and the standards of humane animal care by the criteria outlined in the “Guide for the Care and Use of Laboratory Animals”.

### Blood and tissue sampling

The time points of sampling are shown in Fig. 6 as previously described^12^. Eight randomly selected pups per group (cohorts 1, 2, and 3) were used in subsequent analyses, except for the microarray analysis of cohort 1 (3 randomly selected pups per group) and cohorts 2 and 3 (4 randomly selected pups per group) as well as DNA MBD sequencing (4 randomly selected pups per group in cohort 3). Total lipids were extracted from liver tissue and measured as previously described^12^.

### Microarray analysis

Aliquots (100 ng) of total RNA obtained from 3 animals per group on week 9 (cohort 1) and 4 animals per group on weeks 17 (cohort 2) and 22 (cohort 3) were individually converted to cRNA and labeled with a Gene ChIP® Poly-A RNA Control Kit, WT Amplification Kit, and Gene ChIP® WT Terminal Labeling Kit (Affymetrix, Santa Clara, CA) according to the manufacturer’s instructions. Hybridization, washing, and staining were performed using Affymetrix® MoGene2.1 ST Array Strips and a GeneAtlas® Hybridization Wash and Stain Kit for WT Assay Strips (Affymetrix), according to the manufacturer’s protocols. After washing, MoGene2.1 Array Strips were analyzed using a GeneAtlas Imaging Station (Affymetrix). Data analyses were performed using Expression Console (Affymetrix) and Transcriptome Analysis Console (Affymetrix). The cut-off point: ≤−2 or ≥2 of a linear fold change and ANOVA P values were used as described in our previous studies^45,46^.

### Relative quantitative real-time RT-PCR analysis

The gene expression levels of Cidea and Cidec were measured by relative quantitation in real-time RT-PCR using the Thunderbird SYBR qPCR Mix (TOYOBO, Osaka, Japan). The primers used were purchased from Sigma-Aldrich Japan and were as follows: Cidea; forward primer: CTGTAGCTGTGCCCTGGTTA, reverse primer: CGGGACAGTTCCTGGTCTAT and Cidec; forward primer: ATTCTGAGTCACCCAGGCC, reverse primer: AAATGAGAACAAGAGAGGCAGC.

### Histopathological analysis

Immunohistochemistry was performed as previously described^16^ using the primary antibodies of Cidea (1:400; Novus Biologicals, U.S.A.) and Cidec (1:400; Bioss Antibodies Inc., Massachusetts, U.S.A.). Frozen sections were used for Oil Red O staining. We then measured the mean size (µm²) of lipid droplets (LD) and the mean diameter of each LD using WinRoof ver7.4 software at a scale of a 50-µm magnification. Measurements of 20 randomly selected LD in 4 areas of interest per slide near the central vein were performed in 3.88-mm² fields.

### DNA MBD sequencing

MBD sequencing was performed on cohort 3 only (NN-Veh, NN-TU, UN-Veh, and UN-TU; n = 4 each)^48^. In brief, DNA extracts were sheared to an average length of 200 bp using Covaris S2. Fragment distribution was assessed by the Agilent 2100 Bioanalyzer. The MethylCAp kit (Diagenode, Belgium) was used to enrich methylated fragments from sheared DNA. A library was prepared using the NEBNext® Ultra™ DNA Library Prep Kit for Illumina® (New England Biolabs, Frankfurt am Main, Germany) combined with the NEBNext® Multiplex Oligos for Illumina®-Index Primers Set 1 (New England Biolabs, Frankfurt am Main, Germany). Fragments of approximately 300–450 bp were excised and purified. Paired-end sequencing was performed on Illumina HiSeq4000 PE50 (2×50 bp) and reads were mapped using Bowtie 2 (v2.1.0) software in the sensitive mode. Only concordantly and paired mapped reads located within 400 bp of each other on the mouse reference genome build GRCm38 (mm10) were retained using Bowtie 0.12.7 and peaks were called using MACS14 (v1.4.2). Sites with potential differential methylation were analyzed using DiffBind R-package v2.0.9.

### ChIP assay and quantitative real-time PCR

A ChIP assay was performed on cohort 3 (n = 8 for the 4 groups). Liver tissue samples were homogenized using fixative buffer (1% formaldehyde, 4.5 mM Hepes, pH 8.0, 9 mM NaCl, 0.09 mM EDTA, and 0.04 mM EGTA in phosphate-buffered saline^49^) and incubated at 37 °C for 30 min. The fixation reaction was terminated by the addition of glycine to a final concentration of 1.5 M. After being washed in fluorescence-activated cell sorting solution (2% bovine serum and 0.05% NaN_3_ in PBS), samples were sonicated in SDS lysis buffer (16 mM Tris/HCl, pH 8.0, 10 mM EDTA, 1% SDS, and complete mini) to yield DNA fragments ranging in size between 200 and 500 bp, as confirmed by electrophoresis using a 2% agarose gel. Protein concentrations were adjusted to 2 mg/mL after sonication. ChIP assays were performed using 400 µg of protein in SDS-lysis buffer^50^, and 2 µg each of specific antibodies (Supplementary Table S7). Extracted DNA was then subjected to quantitative PCR using primers corresponding to the indicated sites in the enhancer/promoter and transcribed regions (−2000 bp, −1000 bp, −500 bp, +1 bp, +1000 bp, +2000 bp, +5000 bp, and over +5000 bp) of target genes. Primers for the ChIP assay are listed in Supplementary Table S8. All ChIP signals were calculated by the 2-DDCt method and were normalized to the corresponding input signals as follows: ChIP signals (% input) ¼ 2 (Ct of the input–Ct of the IP sample) × 100, where IP is immunoprecipitation. Notably, the IP of two groups was performed on different days. Non-specific antibody binding to protein-DNA fragments, as indicated by the percentage input using IgG, was negligible (IgG signals <0.1).

### Statistical analysis

Data are expressed as means ± standard deviations (SDs). The significance of differences between two mean values was assessed using the Student’s *t*-test or Mann-Whitney U test as appropriate. The significance of differences among more than four mean values was assessed with the Steel-Dwass test or a one-way ANOVA (unpaired). A *p* value of less than 0.05 was regarded as significant.

## Supplementary information

Dataset 1
