# Supplementary material for: Plasticity of histone modifications around Cidea and Cidec genes with secondary bile in the amelioration of developmentally-programmed hepatic steatosis
Source: Sci Rep. 2019 Nov 19;9:17100. doi: 10.1038/s41598-019-52943-7 (PMC6863835; doi:10.1038/s41598-019-52943-7)
Supplement: Supplementary file 1 — Dataset 1 [file 41598_2019_52943_MOESM1_ESM.pdf]

Plasticity of histone modifications around Cidea and Cidec genes with secondary bile in the amelioration of developmentally-programmed hepatic steatosis

Urmi Jeenat Ferdous[1], Hiroaki Itoh[1], Keiko Muramatsu-Kato[1], Yukiko Kohmura-Kobayashi[1], Natsuyo Hariya[2], Divyanu Jain[1], Naoaki Tamura[1], Toshiyuki Uchida[1], Kazunao Suzuki[1], Yoshihiro Ogawa[3,4], Nobuaki Shiraki[5], Kazuki Mochizuki[6], Takeo Kubota[7], Naohiro Kanayama[1].

[1]Department of Obstetrics and Gynecology, Hamamatsu University School of Medicine, Hamamatsu, Japan;

[2]Department of Nutrition, Faculty of Health and Nutrition, Sciences, Yamanashi Gakuin University, Yamanashi, Japan;

[3]Department of Medicine and Bioregulatory Science, Graduate School of Medical Sciences, Kyushu University, Fukuoka, Japan;

[4]Department of Molecular and Cellular Metabolism, Graduate School of Medical and Dental Sciences, Tokyo Medical and Dental University, Tokyo, Japan;

[5]Department of Life Science and Technology, School of Life Science and Technology, Tokyo Institute of Technology, Yokohama, Japan;

[6]Laboratory of Food and Nutritional Sciences, Department of Local Produce and Food Sciences, Faculty of Life and Environmental Sciences, University of Yamanashi, Yamanashi, Japan;

[7]Faculty of Child Studies, Seitoku University, Matsudo, Japan.

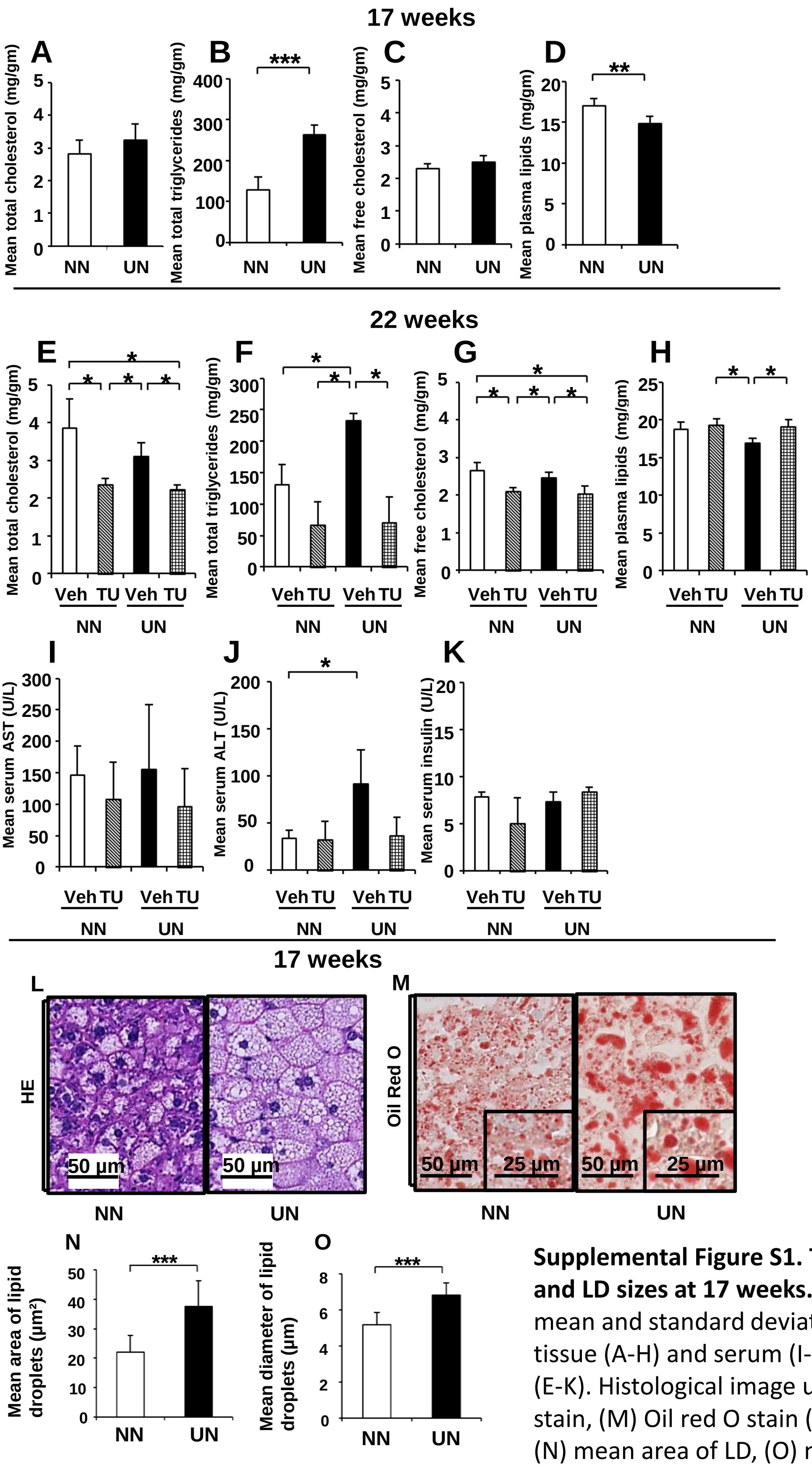

Supplemental Figure S1. Tissue and serum lipid profiles and LD sizes at 17 weeks. Bars and error bars indicate the mean and standard deviations of lipid profiles from liver tissue (A-H) and serum (I-K) in cohort 2 (A-D) and cohort 3 (E-K). Histological image under x40 magnification; (L) HE stain, (M) Oil red O stain (inset with ×80 magnification), (N) mean area of LD, (O) mean diameter of LD with error bars indicating SD. Significance was observed by the Student's t-test (A-D, N, O) and Steel-Dwass test (E-K) (P values were stated as p*<0.05, p**<0.01, and p***<0.001).

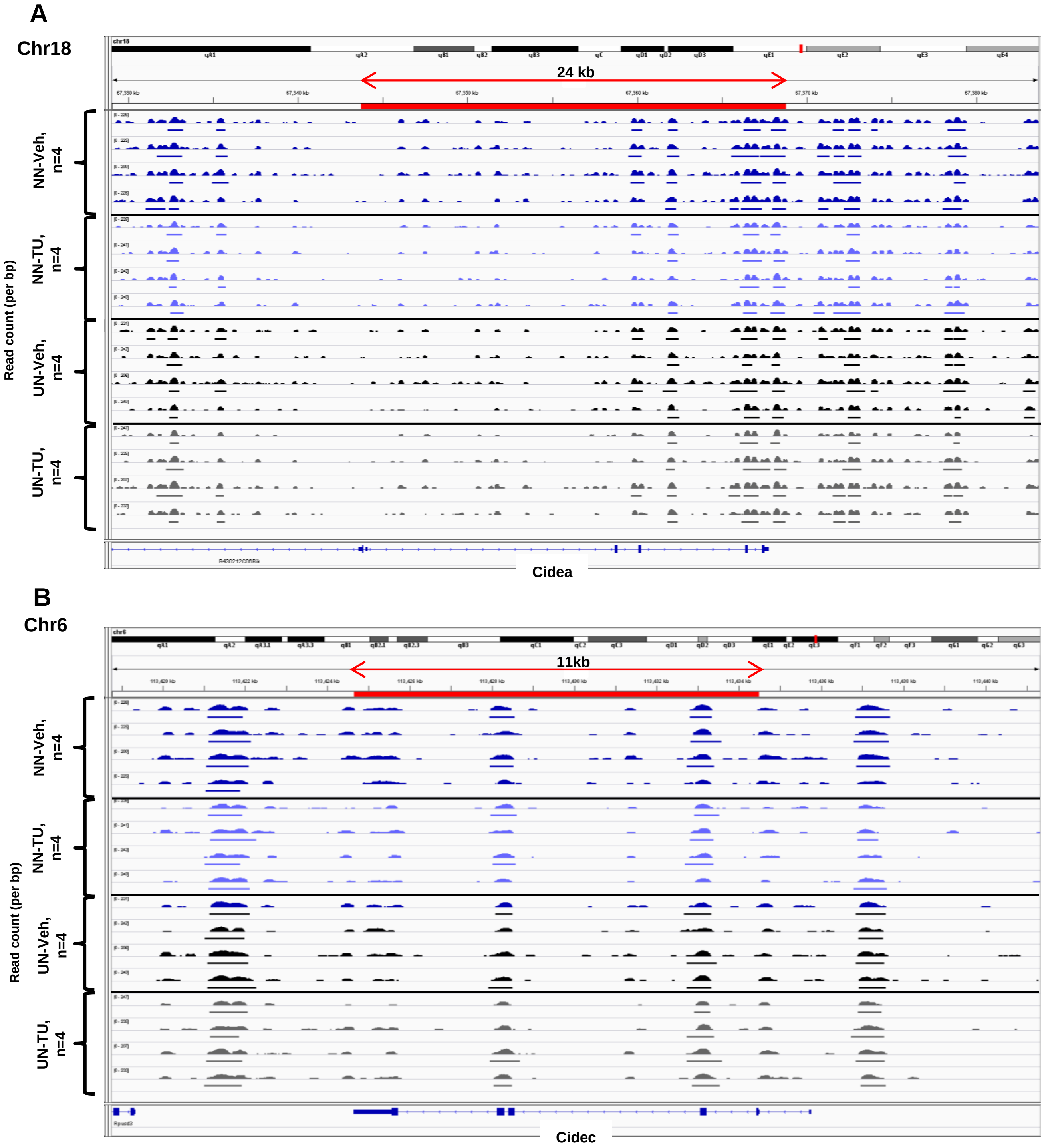

Supplemental Figure S2. Exploratory comparison of MBD-seq data of Cidea and Cidec genes including up- and downstream regions.

A visual comparison of MBD-seq is shown among four groups (NN-Veh; n=4, NN-TU; n=4, UN-Veh; n=4, and UN-TU; n=4). The top row shows the locations of Cidea (A) and Cidec (B) in chromosomes 18 (length of 24 kb) and 6 (length of 11 kb) respectively, and methylation peaks with transcription factor-binding site. Protein coding region marked by red bold line and double arrow. Peaks are color coded as blue, purple, black, and gray for NN-Veh, NN-TU, UN-Veh, and UN-TU, respectively, for clarity. No significant differences were observed in methylation peaks on DNA methylation around entire Cidea gene and entire Cidec gene among the four groups including promoter region.

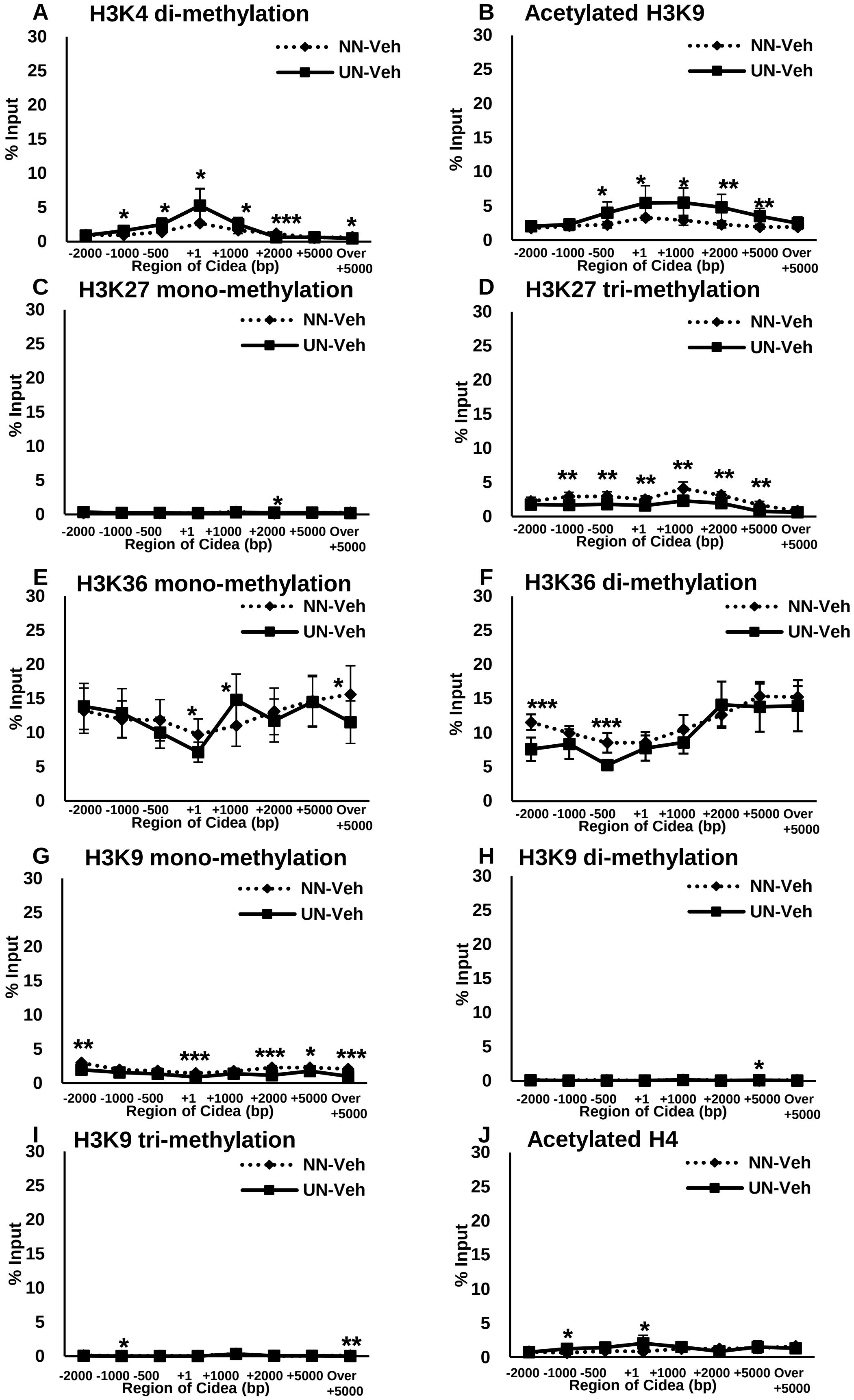

Supplemental Figure S3. Histone modifications around Cidea by ChIP assays in UN *in utero.* Mean values are expressed with SD with error bars (p* <0.05, p**<0.01, p***<0.001), with the Student's t-test or Mann-Whitney U test as appropriate. The X-axis shows the region of gene length and the y-axis is % input.

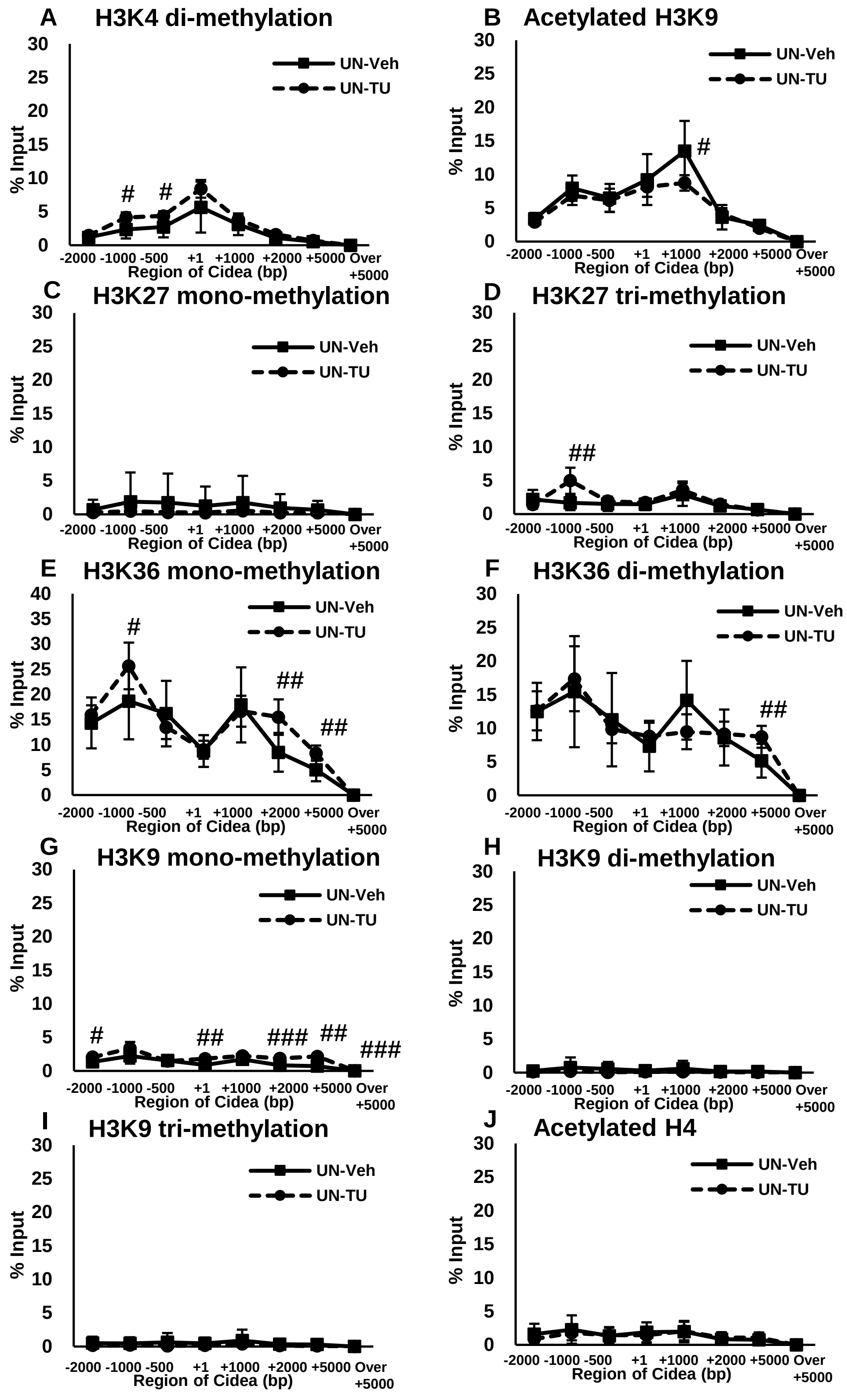

Supplemental Figure S4. Histone modifications around Cidea by ChIP assays in UN *in utero* after the TUDCA treatment. Mean values are expressed with SD with error bars (p# <0.05, p##<0.01, p###<0.001), with the Student's t-test or Mann-Whitney U test as appropriate. The X-axis shows the region of gene length and the y-axis is % input.

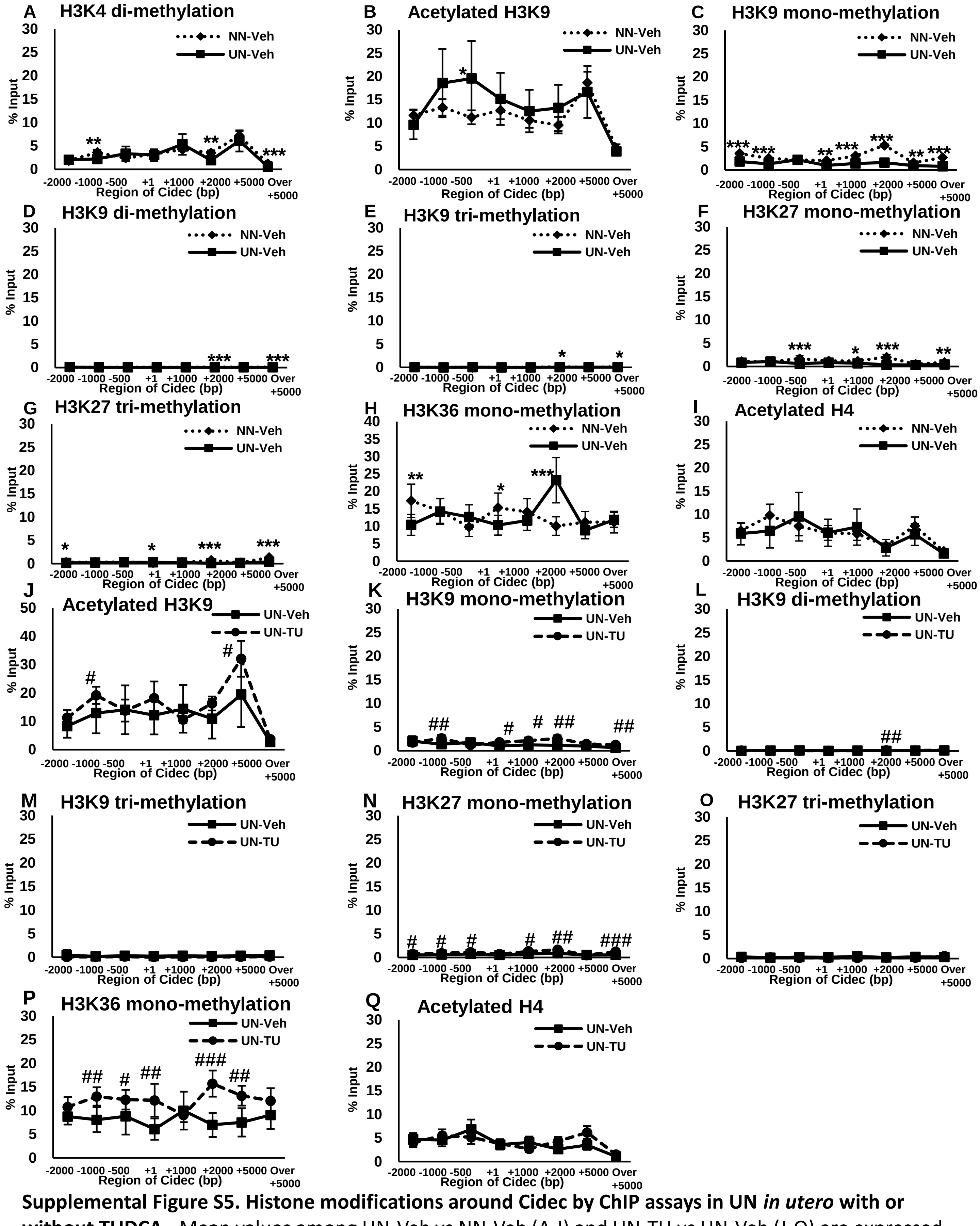

Supplemental Figure S5. Histone modifications around Cidec by ChIP assays in UN *in utero* with or without TUDCA. Mean values among UN-Veh vs NN-Veh (A-I) and UN-TU vs UN-Veh (J-Q) are expressed with SD with error bars (* or # $p<0.05$, ** or ## $p<0.01$, *** or ### $p<0.001$), with the Student's t-test or Mann-Whitney U test as appropriate. The X-axis shows the region of gene length and the y-axis is % input.

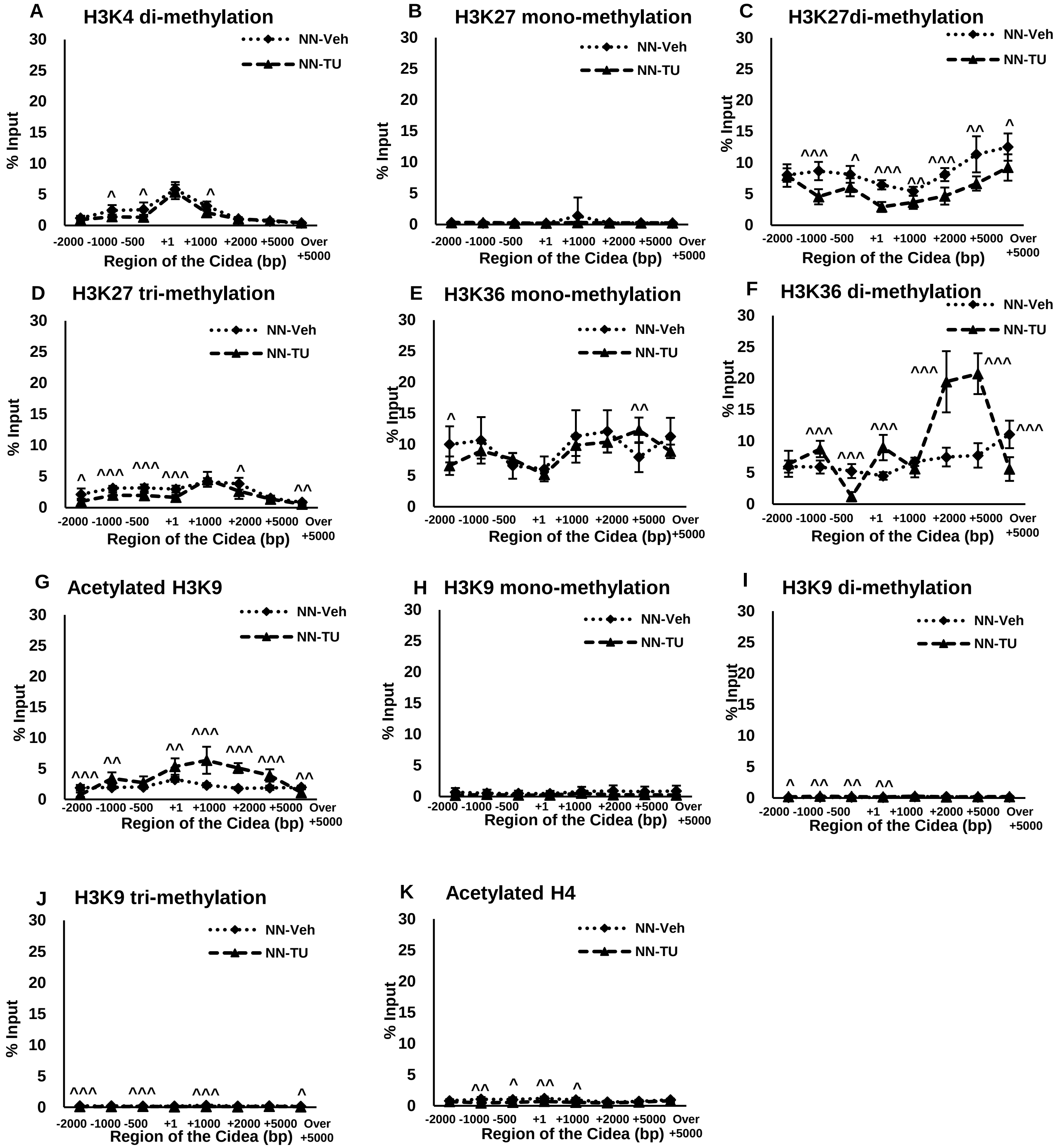

Supplemental Figure S6. Histone modifications around Cidea by ChIP assays in NN *in utero* after the TUDCA treatment. Mean values are expressed with SD with error bars (p^ <0.05, p^^<0.01, p^^^<0.001), with the Student's t-test or Mann-Whitney U test as appropriate. The X-axis shows the region of gene length and the y-axis is % input.

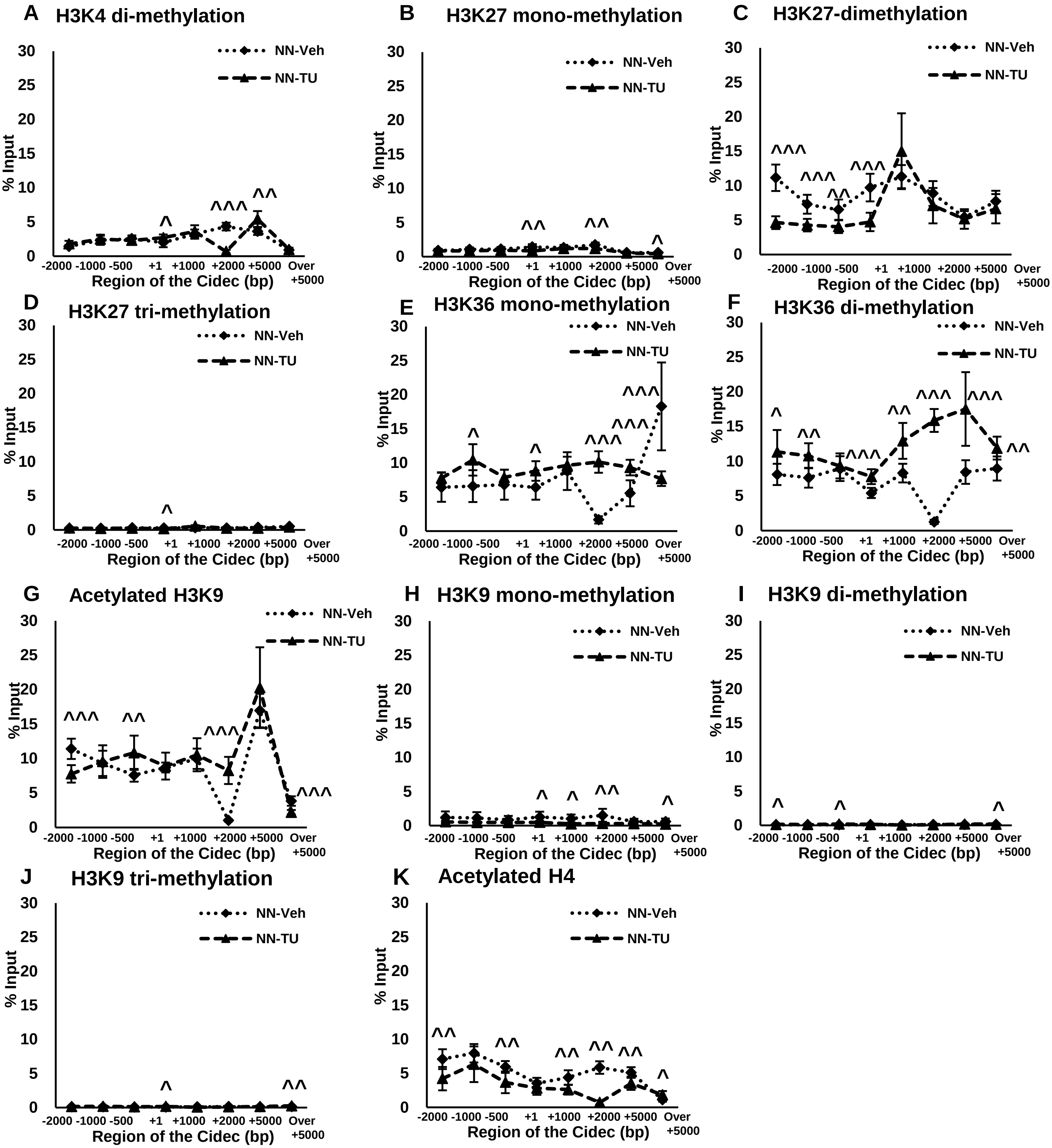

Supplemental Figure S7. Histone modifications around Cidec by ChIP assays in NN *in utero* after the TUDCA treatment. Mean values are expressed with SD with error bars (p^ <0.05, p^^<0.01, p^^^<0.001), with the Student's t-test or Mann-Whitney U test as appropriate. The X-axis shows the region of gene length and the y-axis is % input.

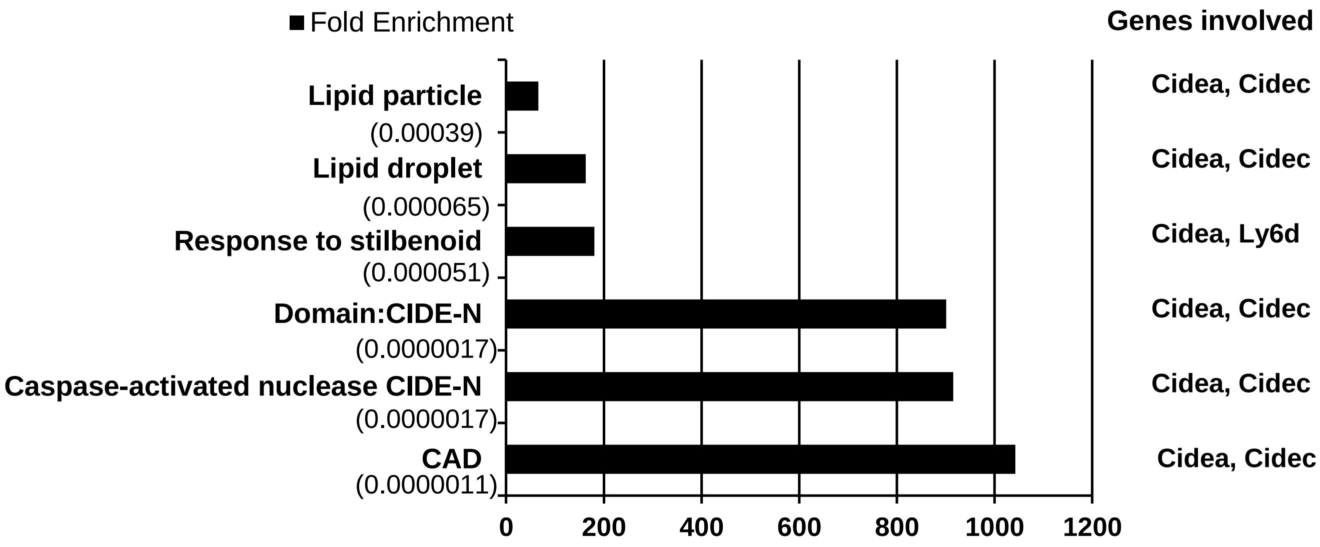

Supplemental Figure S8. Functional Enrichment Analysis of 9 GOI.
X axis showing fold enrichment and y axis showing function involved, along with the genes involved on the right side. We used DAVID Bioinformatics Resources 6.8 for gene enrichment analysis with functional annotation. Statistical significance was calculated by Fisher exact test and mentioned beside each pathway. CAD (Caspase-activated Dnase) or also called CIDE-N (Cell death inducing DNA fragmentation factor-N) is the N terminal part of domain where protein-protein interaction of CIDE proteins (Cidea, Cidec) occur in apoptotic pathway.

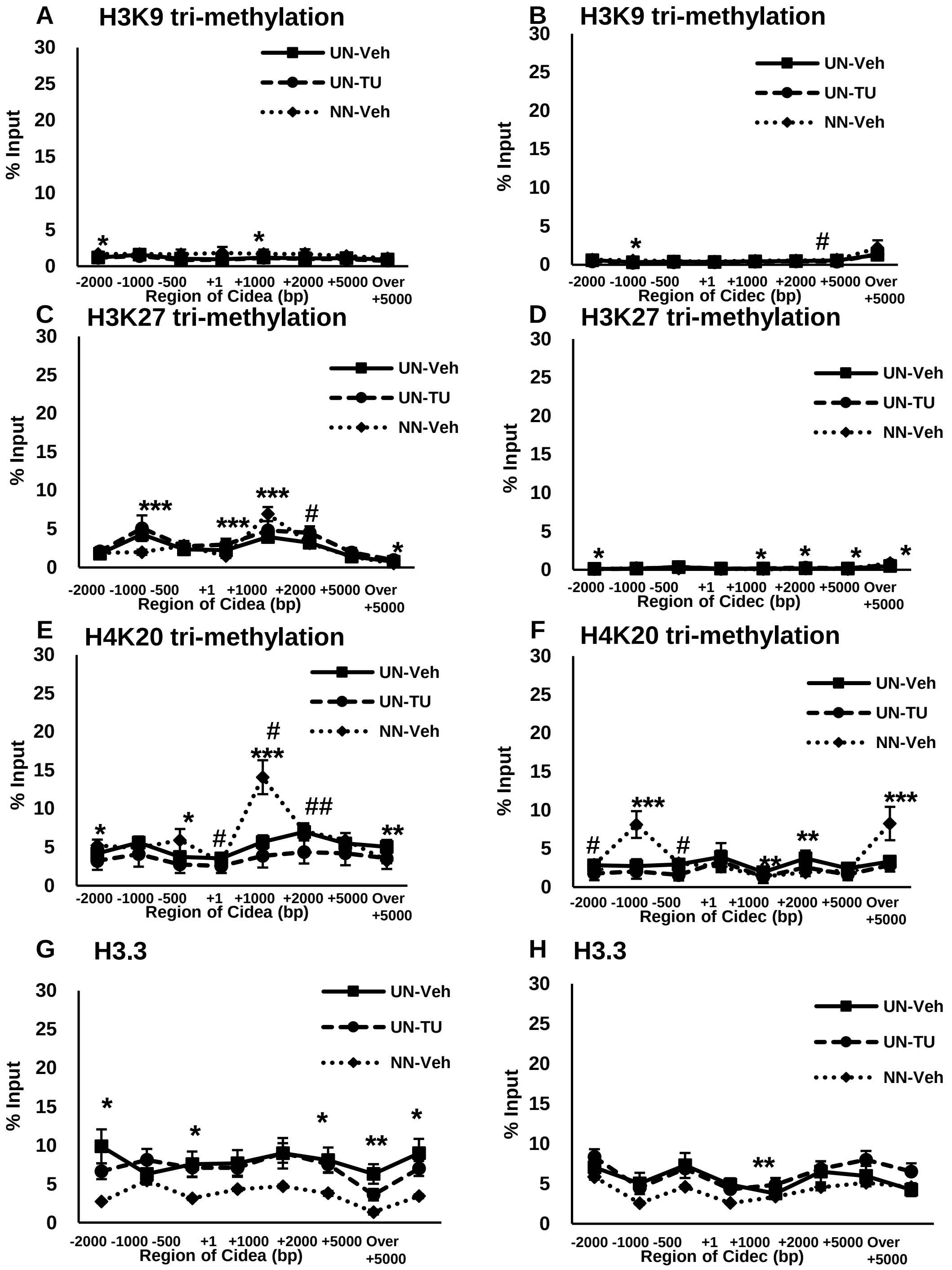

Supplemental Figure S9. Histone modifications around Cidea by ChIP assays at 22 weeks in NN *in utero*, UN *in utero* with or without TUDCA. Mean values are expressed with SD with error bars (* or # p<0.05, ** or ## p<0.01, *** or ### p<0.001), with the Student's t-test or Mann-Whitney U test as appropriate. The X-axis shows the region of gene length and the y-axis is % input. Two different antibodies showed similar negative data in H3K9 (A,B) and H3K27 (C, D) tri-methylation.

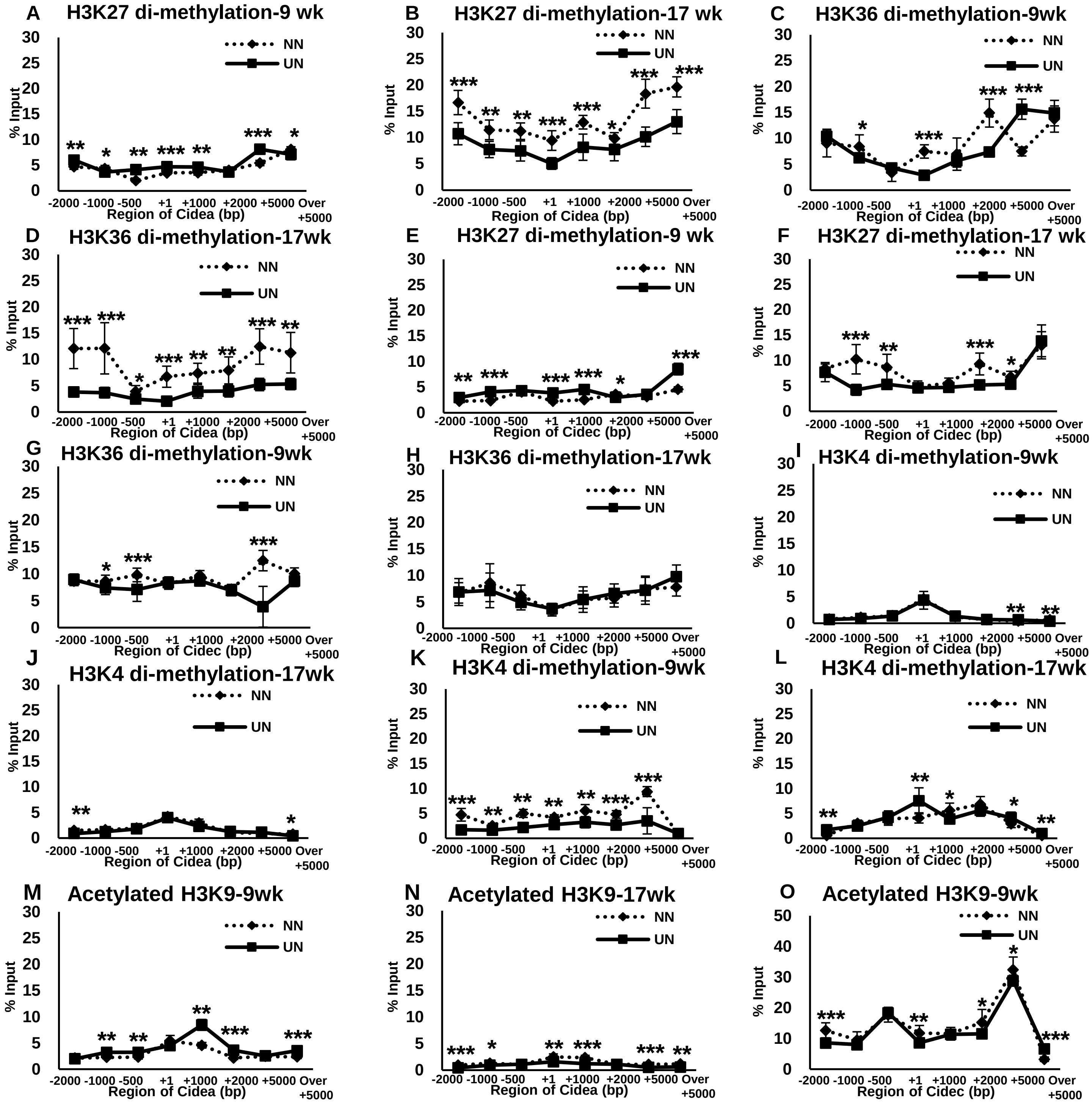

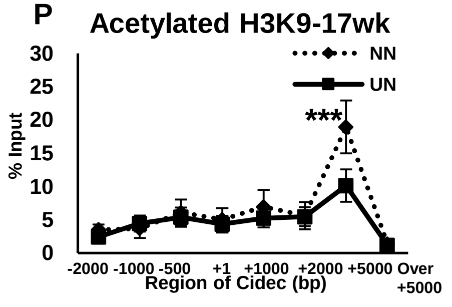

Supplemental Figure S10. Histone modifications around Cidea and Cidec by ChIP assays at 9 weeks and 17 weeks. Mean values among UN vs NN (A-P) are expressed with SD with error bars (* p<0.05, ** p<0.01, *** p<0.001), with the Student's t-test or Mann-Whitney U test as appropriate. The X-axis shows the region of gene length and the y-axis is % input.

Supplemental Table S1. Genes of interest expressed at 9, 17 and 22 weeks of age in a microarray analysis.

A list of genes of interest (GOI) with linear fold changes in mRNA expression in a microarray analysis of cohorts 1, 2, and 3. Comparisons between two groups were performed by a one-way ANOVA between the subjects analyzed (unpaired). The significance of differences is stated as ANOVA P values. In cohorts 1 and 2, genes were selected by filtering genes that were differentially and significantly expressed between UN and NN (cut-off point: ≤-2 or ≥2 linear fold change and ANOVA P value <0.05). In cohort 3, genes that were differentially expressed and then altered by the TUDCA treatment in UN-Veh were selected (cut-off point: ≤-2 or ≥2 linear fold change and ANOVA P value <0.05).

Gene symbol	Linear fold change (UN vs. NN)	ANOVA P value	Bi-weight average signal (NN, log2±SD)	Bi-weight average signal (UN, log2±SD)
**9 weeks**				
Gm24869	2.11	0.02	3.61±0.37	4.69±0.05
Gm17349	2.00	0.02	4.45±0.29	5.45±0.25
Rpl7a-ps8	-2.00	0.01	4.16±0.26	3.16±0.18
Abcd2	-2.05	0.01	8.09±0.20	7.05±0.26
Gm25568	-2.06	0.05	3.46±0.59	2.42±0.09
Gm22935	-2.06	0.02	7.54±0.35	6.50±0.40
Foxq1	-2.21	0.03	7.83±0.57	6.69±0.31
Cish	-3.59	0.03	5.68±0.51	3.84±0.56
**17 weeks**				
Cidea	4.32	0.01	4.28±0.45	6.39±0.86
Acmsd	-3.40	0.03	7.80±0.79	6.03±1.11
Cyp2b13	3.38	0.04	4.56±0.64	6.32±0.89
Orm3	2.91	0.00	3.90±0.45	5.45±0.11
Tcf24	-2.15	0.02	5.15±0.50	4.05±0.46
Gm4956	-2.07	0.00	7.00±0.27	5.95±0.24
Themis	2.70	0.02	4.70±0.31	6.13±0.71
Gm25664	2.34	0.04	2.89±0.11	4.12±0.85
Gm24143	2.30	0.02	5.95±0.45	7.16±0.39
Ifi27l2b	2.29	0.00	4.61±0.45	5.80±0.12
Gm25545	-2.40	0.01	6.10±0.27	4.84±0.66

Gene symbol	Linear fold change (UN vs. NN)	ANOVA P value	Bi-weight average signal (NN, log2±SD)	Bi-weight average signal (UN, log2±SD)
Olfr766	2.28	0.01	2.15±0.07	3.35±0.57
Gm25206	-2.84	0.03	4.92±0.29	3.41±0.96
Klk1b16	2.25	0.03	3.93±0.17	5.11±0.81
Ly6d	2.17	0.00	6.33±0.40	7.45±0.31
Olfr1285	2.14	0.04	2.05±0.07	3.14±0.73
Kifc1	2.13	0.04	2.88±0.35	3.97±0.72
A4gnt	2.13	0.01	2.86±0.04	3.95±0.47
9230110F15Rik	2.12	0.02	2.63±0.34	3.71±0.44
Gm22127	-2.09	0.01	3.63±0.47	2.56±0.43
Vmn1r208; Vmn1r-ps111	2.09	0.01	2.43±0.10	3.49±0.60
Olfr145	2.09	0.04	4.00±0.11	5.06±0.69
Gm25930	-3.30	0.01	5.64±0.30	3.92±0.85
Gm23538	2.03	0.02	2.97±0.29	3.99±0.61
Trav15-1-dv6-1; Trav14-1	2.01	0.03	1.95±0.36	2.96±0.42
Gm24783	2.01	0.05	3.08±0.32	4.08±0.44
Gm24149	-2.98	0.01	5.02±0.45	3.44±0.78
Gm24938; Gm23256; Gm26140; Gm22707; Gm22908; Gm25196	-3.38	0.01	4.75±0.63	2.99±0.75
Gm25985	-2.31	0.01	5.18±0.27	3.97±0.56
Rpp40	-2.00	0.03	5.83±0.39	4.83±0.52
1700054O19Rik	-2.15	0.04	6.44±0.49	5.34±0.73
Snx29	-2.08	0.01	5.24±0.18	4.18±0.40
Snora81; Eif4a2	-2.03	0.05	6.36±0.27	5.34±0.80
Gm25024	-2.16	0.05	3.87±0.48	2.76±0.38
Gm9703	-2.00	0.05	8.63±0.19	7.63±0.84
4932415M13Rik	-2.13	0.00	3.56±0.36	2.47±0.21
Gm22645	-3.02	0.02	4.59±0.58	3.00±0.55
Zfp119a	-2.08	0.02	5.22±0.35	4.16±0.55
Gm24292	-2.03	0.02	3.08±0.61	2.06±0.31
Cyp17a1	-3.38	0.02	9.93±0.50	8.18±0.92
Gm23503	-3.32	0.01	6.56±0.46	4.83±0.83
Gm4450	-2.10	0.04	4.90±0.29	3.83±0.57

Gene symbol	Linear fold change (UN vs. NN)	ANOVA P value	Bi-weight average signal (NN, log2±SD)	Bi-weight average signal (UN, log2±SD)
Hsd3b4; Gm10681; Gm4450	-2.79	0.00	6.25±0.58	4.77±0.14
Hsd3b4; Gm10681; Gm4450	-2.79	0.00	6.25±0.58	4.77±0.14
Hsd3b5	-17.71	0.00	7.19±1.07	3.04±0.73
Gm12508	-2.37	0.04	6.37±0.09	5.12±0.94
Gm25937	-3.19	0.02	5.98±0.40	4.30±0.95
Gm4963	-3.19	0.04	5.86±0.29	4.19±1.23
Spr-ps1	-2.11	0.00	4.95±0.07	3.88±0.38
Clec2h	-2.59	0.01	6.07±0.66	4.70±0.29
Gm22155	-3.15	0.03	6.71±0.79	5.06±0.99
Slco1a1	-2.91	0.01	9.62±0.51	8.08±1.07
Gm24701	-5.47	0.03	5.91±0.82	3.46±1.10
Gm24938; Gm23256; Gm26140; Gm22707; Gm22908	-3.38	0.01	4.75±0.63	2.99±0.75
Hamp2	-2.39	0.03	9.80±0.21	8.54±1.18
Cdh1	-2.01	0.00	6.47±0.32	5.46±0.16
Slc10a2	-2.22	0.03	7.21±0.30	6.06±0.82
Snord13	-2.70	0.05	7.26±0.28	5.83±1.07
Gm23128	-2.13	0.01	4.37±0.20	3.28±0.44
Gm10717	-2.27	0.05	8.22±0.22	7.04±1.38
Gm22717	-2.16	0.01	4.67±0.16	3.56±0.63
Gm22866	-5.12	0.04	9.10±0.53	6.74±1.81
mt-Tt	-3.51	0.01	5.66±0.24	3.85±0.97
mt-Ta	-3.59	0.00	8.33±0.37	6.49±0.82
mt-Tc	-3.93	0.00	6.27±0.70	4.29±0.46
mt-Ty	-5.07	0.01	6.53±0.79	4.19±0.98
mt-Ts1	-4.37	0.03	7.00±0.55	4.87±1.34
mt-Tc	-3.93	0.00	6.27±0.70	4.29±0.46
mt-Ty	-5.04	0.01	6.55±0.74	4.22±0.94
mt-Ts1	-5.53	0.01	6.43±0.62	3.96±1.21
mt-Tl2	-2.79	0.01	5.71±0.26	4.23±1.03
mt-Tt	-2.78	0.00	4.78±0.23	3.30±0.38
mt-Ta	-3.08	0.01	8.32±0.36	6.69±0.92

Gene symbol	Linear fold change (UN vs. NN)	ANOVA P value	Bi-weight average signal (NN, log2±SD)	Bi-weight average signal (UN, log2±SD)
**22 weeks**				
Cidea	4.47±0.38	4.77±0.77	7.99±0.41	5.45±1.25
Cidec	6.47±0.62	6.40±0.78	8.96±0.17	6.96±1.36
Rgs16	7.14±0.72	7.91±1.14	9.31±0.82	7.23±1.84
Orm3	4.23±0.54	3.89±0.17	5.97±0.41	3.83±0.12
Igfbp1	7.38±0.17	7.45±0.54	6.34±0.12	7.55±0.52
Rarres1	5.59±0.14	6.47±1.11	7.22±0.19	5.98±0.89
Ly6d	6.04±0.61	5.96±0.35	7.62±0.56	6.34±0.34
Sprr1a	3.78±0.25	3.43±0.30	5.04±0.40	3.79±0.58
Acot2	7.69±0.24	7.75±0.25	8.91±0.24	7.81±0.89
Slc3a1	9.84±0.20	9.05±0.56	8.43±0.02	9.44±0.73
Themis	4.61±0.36	3.50±0.26	5.82±0.25	3.95±0.38
Cyp17a1	10.2±0.15	8.46±1.06	8.83±0.47	10.36±0.52
Gstm3	7.98±0.56	7.05±0.39	9.13±0.16	7.78±0.28
Wee1	5.44±0.11	5.04±0.57	6.53±0.52	5.11±0.49
Ifi27l2b	4.74±0.57	4.48±0.22	5.81±0.40	4.52±0.38

Supplementary Table S2. Candidate gene from expression analysis in cohort 3

NN-Veh, NN-TU, UN-Veh, and UN-TU. Genes in UN-Veh were differentially expressed from those in NN-Veh, and restored by the TUDCA treatment in UN-TU from those in NN-TU (cut-off point: ≤-2 or ≥2 linear fold change and ANOVA P-value <0.05).

Gene Symbol	Effects of UN *in utero* (UN-Veh vs. NN-Veh)	ANOVA P value (UN-Veh vs. NN-Veh)	TUDCA response in NN (NN-TU vs. NN-Veh	ANOVA P value (NN-TU vs. NN-Veh	TUDCA response in UN (UN-TU vs. UN-Veh)	ANOVA P value (UN-TU vs. UN-Veh)
Cidea	11.49	0.000	1.23	0.54	-5.82	0.01
Cidec	5.60	0.000	-1.05	0.70	-3.99	0.02
Rgs16	4.51	0.00	1.71	0.11	-4.24	0.10
Orm3	3.35	0.00	-1.26	0.33	-4.39	0.000
Rarres1	3.11	0.000	1.85	0.53	-2.36	0.01
Ly6d	2.98	0.000	-1.06	0.47	-2.42	0.03
Sprr1a	2.40	0.02	-1.27	0.38	-2.38	0.01
Acot2	2.33	0.00	1.05	0.18	-2.13	0.02
Themis	2.31	0.000	-2.17	0.71	-3.66	0.05
Gstm3	2.22	0.000	-1.90	0.08	-2.56	0.02
Wee1	2.11	0.00	-1.33	0.00	-2.67	0.000
Ifi27l2b	2.09	0.00	-1.20	0.07	-2.44	0.01
Igfbp1	-2.06	0.01	1.05	0.06	2.31	0.000
Cyp17a1	-2.58	0.01	-3.34	0.19	2.89	0.00
Slc3a1	-2.67	0.04	-1.73	0.21	2.02	0.01

Supplemental Table S3. Longitudinal gene expression analysis (comparison between cohorts) to investigate candidate genes of interest over 9, 17, and 22 weeks

Linear fold changes in different genes shown with the longitudinal time periods of cohorts 1, 2, and 3 with their ANOVA P values. Here we observed 133 genes altered in their expression in cohort 2 vs cohort 1 (cut-off point: ≤-2 or ≥2 linear fold change, ANOVA P value <0.05) among UN *in utero* and contrast them against genes among NN (cohort 2 vs cohort 1).

Gene Symbol	Linear Fold Change (UN in cohort 2 vs cohort 1)	ANOVA P value (UN in cohort 2 vs cohort 1)	Linear Fold Change (NN in cohort 2 vs cohort 1)	ANOVA P value (NN in cohort 2 vs cohort 1)	Linear Fold Change (UN in cohort 3 vs cohort 2)	ANOVA P value (UN in cohort 3 vs cohort 2)	Linear Fold Change (In cohort 3; UN-Veh vs. NN-Veh)	ANOVA P value (In cohort 3; UN-Veh vs. NN-Veh)	Linear Fold Change (In cohort 3; UN-TU vs. UN-Veh)	ANOVA P value (In cohort 3; UN-TU vs. UN-Veh)
Cyp2b9	40.81	0.003	7.88	0.037	1.22	0.281	1.67	0.001	1.10	0.688
Rgs16	9.67	0.000	5.71	0.439	1.55	0.218	4.51	0.004	-4.24	0.097
Saa2	9.41	0.036	4.31	0.009	-1.23	0.594	1.47	0.109	-1.46	0.095
Cidec	8.77	0.001	2.87	0.071	1.64	0.162	5.60	0.000	-3.99	0.017
Apoa4	6.39	0.015	3.33	0.004	1.26	0.160	2.65	0.000	-1.70	0.029
Cidea	5.68	0.010	1.62	0.260	3.04	0.009	11.49	0.000	-5.82	0.011
Slc22a27	5.37	0.032	6.28	0.007	1.73	0.249	2.28	0.006	-1.66	0.054
Lcn2	5.29	0.012	1.97	0.001	1.20	0.809	1.77	0.052	-1.44	0.358
Themis	5.09	0.003	2.41	0.001	-1.24	0.453	2.31	0.003	-3.66	0.000
Lgals1	4.89	0.016	3.69	0.001	-1.24	0.777	1.61	0.018	-1.73	0.016
Ifi27l2b	4.06	0.000	2.49	0.005	1.01	0.885	2.09	0.037	-2.44	0.006
Cyp17a1	4.04	0.018	7.63	0.004	1.57	0.207	-2.58	0.002	2.89	0.009
Cyp2b13	3.64	0.019	1.38	0.100	1.39	0.331	2.05	0.023	1.14	0.486
9030619 P08Rik	3.43	0.004	1.45	0.099	1.29	0.183	3.26	0.000	-1.16	0.316
Sprr1a	3.09	0.022	1.73	0.169	1.04	0.806	2.40	0.002	-2.38	0.020
LOC1026 34708; Gm11695	3.09	0.005	2.71	0.074	-1.16	0.908	-1.12	0.931	-1.66	0.027

Gene Symbol	Linear Fold Change (UN in cohort 2 vs cohort 1)	ANOVA P value (UN in cohort 2 vs cohort 1)	Linear Fold Change (NN in cohort 2 vs cohort 1)	ANOVA P value (NN in cohort 2 vs cohort 1)	Linear Fold Change (UN in cohort 3 vs cohort 2)	ANOVA P value (UN in cohort 3 vs cohort 2)	Linear Fold Change (In cohort 3; UN-Veh vs. NN-Veh)	ANOVA P value (In cohort 3; UN-Veh vs. NN-Veh)	Linear Fold Change (In cohort 3; UN-TU vs. UN-Veh)	ANOVA P value (In cohort 3; UN-TU vs. UN-Veh)
LOC102635290; Gm12750	3.05	0.000	1.25	0.027	-1.15	0.346	2.02	0.001	-1.87	0.001
Anxa2	2.69	0.005	1.79	0.011	1.15	0.388	1.44	0.087	-1.56	0.023
Ly6d	2.64	0.001	1.30	0.116	1.12	0.817	2.98	0.021	-2.42	0.008
Mogat1	2.63	0.042	1.77	0.006	1.29	0.433	1.97	0.003	-1.46	0.021
Slc16a5	2.59	0.016	2.40	0.002	1.32	0.168	1.26	0.037	-1.97	0.004
Orm3	2.53	0.000	1.10	0.501	1.44	0.079	3.35	0.002	-4.39	0.000
Olfr373	2.44	0.035	-1.16	0.578	-1.56	0.149	1.02	0.960	-1.33	0.024
2010003K11Rik	2.32	0.003	1.57	0.082	1.41	0.074	2.01	0.005	-1.75	0.020
Olfr766	2.30	0.024	-1.19	0.130	-2.22	0.096	-1.08	0.643	1.03	0.459
Mmp12	2.23	0.008	1.28	0.101	-1.21	0.363	1.14	0.083	1.02	0.898
Lamb3	2.20	0.039	1.78	0.020	1.04	0.885	1.32	0.034	-1.48	0.015
Gprc5b	2.15	0.023	1.20	0.212	1.27	0.456	1.23	0.327	1.37	0.693
Cfd	2.14	0.020	1.53	0.075	-1.01	0.861	1.32	0.034	-1.59	0.009
Mtnr1a	2.14	0.046	1.86	0.026	1.25	0.153	1.31	0.013	-1.01	0.712
Osbpl3	2.14	0.011	1.86	0.031	1.44	0.093	1.85	0.001	-1.97	0.013
Gnpda1	2.11	0.011	2.07	0.004	1.07	0.203	1.04	0.279	-1.15	0.103
Spc24	2.07	0.005	1.43	0.008	1.00	0.880	1.38	0.047	-2.12	0.005
Enc1	2.05	0.002	1.22	0.126	1.01	0.374	1.40	0.013	-1.28	0.005
Gm10680	2.04	0.004	1.31	0.030	1.09	0.633	1.66	0.016	-1.70	0.052
Cenpn	2.04	0.004	1.23	0.146	-1.41	0.049	-1.07	0.137	-1.04	0.531
Gm23538	2.04	0.044	-1.29	0.182	-1.56	0.091	1.04	0.730	-1.22	0.099
Gm11788;LOC102640850	2.01	0.027	1.47	0.003	1.10	0.440	1.13	0.145	-1.31	0.043
Gm26159	-2.00	0.006	-1.47	0.231	1.26	0.056	-1.10	0.854	-1.05	0.784
Gm25107	-2.02	0.018	-1.44	0.244	-1.37	0.366	-1.22	0.329	1.75	0.021
Gm22785	-2.03	0.031	-1.26	0.279	1.86	0.021	1.09	0.402	-1.20	0.608
Gm23422	-2.04	0.001	-1.10	0.725	1.60	0.002	1.16	0.451	-1.06	0.207

Gene Symbol	Linear Fold Change (UN in cohort 2 vs cohort 1)	ANOVA P value (UN in cohort 2 vs cohort 1)	Linear Fold Change (NN in cohort 2 vs cohort 1)	ANOVA P value (NN in cohort 2 vs cohort 1)	Linear Fold Change (UN in cohort 3 vs cohort 2)	ANOVA P value (UN in cohort 3 vs cohort 2)	Linear Fold Change (In cohort 3; UN-Veh vs. NN-Veh)	ANOVA P value (In cohort 3; UN-Veh vs. NN-Veh)	Linear Fold Change (In cohort 3; UN-TU vs. UN-Veh)	ANOVA P value (In cohort 3; UN-TU vs. UN-Veh)
Gm8430	-2.04	0.015	1.06	0.769	1.30	0.081	-1.32	0.200	1.01	0.793
Apom	-2.05	0.002	-1.75	0.000	-1.05	0.957	-1.04	0.218	1.28	0.017
Tcf24; Gm10567	-2.05	0.003	-1.18	0.090	1.20	0.356	-1.52	0.004	1.27	0.213
Mir1970	-2.06	0.009	-1.19	0.152	1.87	0.011	-1.24	0.223	-1.07	0.920
Gm16551 ; Gm20649	-2.07	0.004	-1.42	0.034	-1.05	0.982	-1.01	0.768	1.13	0.129
mt-Ta	-2.07	0.034	-1.07	0.659	1.92	0.041	-1.11	0.454	1.38	0.236
Snord52; 1110038 B12Rik	-2.09	0.036	-1.39	0.695	1.19	0.351	1.03	0.767	1.02	0.352
Gm24202	-2.09	0.031	1.74	0.011	1.54	0.270	1.26	0.692	-1.63	0.041
Zbtb16	-2.12	0.042	-1.33	0.561	-1.11	0.535	-3.42	0.000	-1.02	0.471
Gm2788	-2.12	0.028	-2.71	0.079	1.58	0.117	2.13	0.002	-1.23	0.699
n-R5s127	-2.13	0.020	-1.80	0.092	-1.02	0.605	-1.13	0.823	1.31	0.898
Rpl36-ps3	-2.14	0.020	-1.26	0.243	1.42	0.174	1.06	0.752	-1.06	0.987
Gm22771	-2.14	0.045	-1.35	0.190	1.60	0.171	1.28	0.125	-1.10	0.584
Gm26109	-2.15	0.006	-1.87	0.107	1.36	0.097	-1.10	0.690	1.07	0.588
n-R5s64	-2.16	0.005	-1.79	0.037	1.32	0.045	-1.13	0.491	1.06	0.704
Gm23247	-2.19	0.049	-1.25	0.038	2.35	0.021	-1.16	0.181	-1.07	0.953
Cyp26a1	-2.22	0.001	-1.24	0.444	1.94	0.009	1.35	0.269	-1.28	0.965
Gm4956	-2.23	0.001	-1.03	0.504	1.24	0.033	-1.73	0.001	1.41	0.155
Eif4ebp3	-2.24	0.003	-1.23	0.161	1.05	0.400	-1.08	0.256	-1.00	0.958
Npas2	-2.25	0.001	-2.03	0.150	1.60	0.001	-1.25	0.171	2.36	0.032
Fgfr2	-2.25	0.029	-1.03	0.692	1.74	0.052	-1.29	0.015	1.28	0.044
4833411 C07Rik	-2.28	0.005	-1.51	0.019	1.31	0.069	1.20	0.225	1.03	0.764
mt-Ta	-2.29	0.028	-1.08	0.675	2.13	0.022	-1.21	0.396	1.47	0.210
Wrnip1	-2.30	0.023	-1.36	0.010	1.47	0.059	-1.14	0.066	1.23	0.001
Irf6	-2.31	0.008	-2.17	0.000	1.07	0.318	-1.18	0.167	1.26	0.016

Gene Symbol	Linear Fold Change (UN in cohort 2 vs cohort 1)	ANOVA P value (UN in cohort 2 vs cohort 1)	Linear Fold Change (NN in cohort 2 vs cohort 1)	ANOVA P value (NN in cohort 2 vs cohort 1)	Linear Fold Change (UN in cohort 3 vs cohort 2)	ANOVA P value (UN in cohort 3 vs cohort 2)	Linear Fold Change (In cohort 3; UN-Veh vs. NN-Veh)	ANOVA P value (In cohort 3; UN-Veh vs. NN-Veh)	Linear Fold Change (In cohort 3; UN-TU vs. UN-Veh)	ANOVA P value (In cohort 3; UN-TU vs. UN-Veh)
Gm25683	-2.33	0.022	-1.31	0.325	1.49	0.066	1.06	0.824	-1.38	0.004
Ndrg1	-2.35	0.009	-2.67	0.002	1.48	0.020	-1.06	0.357	1.41	0.004
Adck5	-2.40	0.026	-1.88	0.001	1.64	0.076	-1.06	0.868	1.03	0.511
Gm26216	-2.40	0.034	-1.08	0.550	1.63	0.023	1.40	0.077	-1.22	0.335
mt-Ty	-2.43	0.044	-1.18	0.878	2.67	0.042	-1.19	0.935	1.31	0.369
Gm26293	-2.44	0.027	-1.30	0.026	1.39	0.329	1.36	0.338	-1.15	0.278
Gm23579	-2.46	0.047	-1.02	0.638	1.33	0.216	-1.22	0.711	-1.11	0.237
Cyp1a2	-2.48	0.046	-2.09	0.007	1.04	0.278	-1.12	0.189	1.14	0.244
Rps15a-ps5	-2.51	0.033	-1.26	0.355	1.65	0.132	-1.11	0.333	-1.08	0.602
Gm25930	-2.52	0.037	1.63	0.042	2.23	0.048	-1.16	0.227	1.08	0.846
Gm22270	-2.54	0.012	-2.30	0.151	1.15	0.883	-1.13	0.294	1.15	0.244
Gm24869	-2.55	0.006	1.10	0.861	1.47	0.033	1.17	0.182	-1.24	0.035
Adh6-ps1	-2.57	0.041	-1.45	0.267	1.01	0.323	-1.34	0.029	1.34	0.079
Idi1; Gm13502	-2.63	0.043	-1.80	0.045	1.44	0.141	1.46	0.104	2.19	0.002
Fabp5; Gm6166	-2.64	0.005	-3.11	0.029	1.22	0.422	-1.05	0.589	1.30	0.073
Fabp5; Gm6166	-2.77	0.009	-2.52	0.024	1.28	0.412	-1.02	0.555	1.26	0.075
Fbxo21	-2.77	0.004	-2.74	0.003	1.70	0.016	1.17	0.207	1.03	0.802
Gm26330; LOC101055915; Gm15501; Rps8	-2.82	0.014	-1.51	0.026	1.64	0.097	-1.12	0.244	1.01	0.719
Gm23344	-2.86	0.048	-1.20	0.838	2.40	0.038	1.02	0.838	-1.15	0.378
Serpina1e	-2.87	0.013	-1.35	0.126	1.06	0.327	-1.86	0.002	1.71	0.015
Gm23650; Nop56	-2.92	0.017	-1.65	0.044	-1.12	0.538	1.07	0.992	1.11	0.092
Slco2a1	-2.92	0.024	-2.10	0.005	-1.23	0.832	-1.41	0.004	1.86	0.004

Gene Symbol	Linear Fold Change (UN in cohort 2 vs cohort 1)	ANOVA P value (UN in cohort 2 vs cohort 1)	Linear Fold Change (NN in cohort 2 vs cohort 1)	ANOVA P value (NN in cohort 2 vs cohort 1)	Linear Fold Change (UN in cohort 3 vs cohort 2)	ANOVA P value (UN in cohort 3 vs cohort 2)	Linear Fold Change (In cohort 3; UN-Veh vs. NN-Veh)	ANOVA P value (In cohort 3; UN-Veh vs. NN-Veh)	Linear Fold Change (In cohort 3; UN-TU vs. UN-Veh)	ANOVA P value (In cohort 3; UN-TU vs. UN-Veh)
Snora64; Gm8842; Gm8841; Gm10420; Gm6139; Rps2	-2.98	0.012	-2.10	0.147	-1.09	0.688	-1.30	0.133	1.30	0.169
Scarna6	-3.01	0.043	-1.08	0.405	-1.04	0.869	-1.69	0.003	1.27	0.085
Tcf24	-3.07	0.007	-1.15	0.224	-1.08	0.721	-1.94	0.014	1.65	0.213
C8b	-3.08	0.050	-1.81	0.002	-1.39	0.621	-2.34	0.000	1.62	0.028
Gm25985	-3.11	0.011	-1.35	0.135	1.68	0.064	1.03	0.330	1.28	0.992
Snora15	-3.17	0.039	-1.91	0.150	1.59	0.369	-1.16	0.190	1.04	0.187
Gm22155	-3.20	0.026	1.35	0.616	1.44	0.244	-2.17	0.056	1.19	0.761
Smad9	-3.33	0.019	-1.48	0.034	1.02	0.844	1.27	0.032	-1.14	0.221
Cyp3a41b; Cyp3a41a; Cyp3a11; Cyp3a16; Cyp3a44	-3.34	0.015	-3.87	0.000	1.44	0.120	1.54	0.009	-1.55	0.019
Cyp3a41b; Cyp3a41a; Cyp3a11; Cyp3a16; Cyp3a44	-3.40	0.015	-3.99	0.000	1.49	0.111	1.57	0.009	-1.59	0.018
Snord12; 1500012F01Rik	-3.44	0.032	1.12	0.711	2.12	0.095	1.10	0.716	-1.19	0.439
Acmsd	-3.45	0.026	1.02	0.729	1.14	0.370	-3.57	0.000	1.63	0.124

Gene Symbol	Linear Fold Change (UN in cohort 2 vs cohort 1)	ANOVA P value (UN in cohort 2 vs cohort 1)	Linear Fold Change (NN in cohort 2 vs cohort 1)	ANOVA P value (NN in cohort 2 vs cohort 1)	Linear Fold Change (UN in cohort 3 vs cohort 2)	ANOVA P value (UN in cohort 3 vs cohort 2)	Linear Fold Change (In cohort 3; UN-Veh vs. NN-Veh)	ANOVA P value (In cohort 3; UN-Veh vs. NN-Veh)	Linear Fold Change (In cohort 3; UN-TU vs. UN-Veh)	ANOVA P value (In cohort 3; UN-TU vs. UN-Veh)
Cyp2c54	-3.52	0.031	-2.18	0.127	-1.15	0.519	-1.64	0.020	1.30	0.162
Snord13	-3.56	0.036	-1.58	0.249	1.36	0.707	1.22	0.812	-1.23	0.731
Cyp4a12a	-3.62	0.006	-1.98	0.109	-1.46	0.546	-1.14	0.564	1.19	0.855
Avpr1a	-3.66	0.010	-2.88	0.003	1.20	0.391	-1.67	0.002	2.47	0.005
Gm4450	-3.71	0.010	-1.15	0.156	1.28	0.533	-1.22	0.124	1.20	0.150
Igfbp2	-3.75	0.002	-3.82	0.002	1.53	0.040	1.01	0.787	-1.39	0.002
Gm24938;Gm23256;Gm26140;Gm22707;Gm22908;Gm25196	-3.78	0.009	-1.31	0.175	2.68	0.043	-1.25	0.320	1.23	0.384
Gm24938;Gm23256;Gm26140;Gm22707;Gm22908;Gm25196	-3.78	0.009	-1.31	0.175	2.68	0.043	-1.25	0.320	1.23	0.384
Gm24938;Gm23256;Gm26140;Gm22707;	-3.78	0.009	-1.31	0.175	2.68	0.043	-1.25	0.320	1.23	0.384
Chka	-4.19	0.035	-1.63	0.254	2.07	0.027	-1.92	0.004	1.61	0.019
Gm22036	-4.37	0.013	-2.49	0.012	-1.16	0.575	1.01	0.599	1.35	0.124
Gm15998	-4.38	0.003	-2.16	0.018	1.53	0.022	1.02	0.370	2.03	0.001
Gm15611	-4.40	0.012	-5.41	0.001	-1.06	0.872	-1.34	0.223	1.24	0.230
Cyp4a12b	-4.47	0.003	-1.95	0.130	-1.41	0.552	-1.48	0.003	-1.05	0.531

Gene Symbol	Linear Fold Change (UN in cohort 2 vs cohort 1)	ANOVA P value (UN in cohort 2 vs cohort 1)	Linear Fold Change (NN in cohort 2 vs cohort 1)	ANOVA P value (NN in cohort 2 vs cohort 1)	Linear Fold Change (UN in cohort 3 vs cohort 2)	ANOVA P value (UN in cohort 3 vs cohort 2)	Linear Fold Change (In cohort 3; UN-Veh vs. NN-Veh)	ANOVA P value (In cohort 3; UN-Veh vs. NN-Veh)	Linear Fold Change (In cohort 3; UN-TU vs. UN-Veh)	ANOVA P value (In cohort 3; UN-TU vs. UN-Veh)
Snord68; Gm10071 ; Rpl13	-4.57	0.034	1.18	0.586	1.76	0.185	1.08	0.406	-1.05	0.354
Cdh1	-4.64	0.000	-2.10	0.038	1.00	0.874	-1.42	0.027	1.67	0.019
Ces2a	-4.73	0.013	-2.97	0.000	1.42	0.248	-1.21	0.003	-1.20	0.003
Clec2h	-4.76	0.011	-2.79	0.071	1.02	0.982	-1.25	0.099	-1.57	0.005
Csad	-5.24	0.001	-2.68	0.000	1.43	0.140	-1.05	0.769	-1.33	0.278
Gm10717	-5.27	0.047	-1.75	0.117	4.82	0.036	1.81	0.245	-1.08	0.919
Slco1a1	-5.85	0.005	-1.76	0.030	-3.00	0.199	-2.84	0.063	6.27	0.005
Cyp3a11	-6.41	0.000	-5.87	0.000	1.72	0.037	1.66	0.020	-1.65	0.044
Hsd3b4; Gm10681 ; Gm4450	-6.82	0.000	-2.47	0.025	-1.10	0.152	-1.38	0.077	1.19	0.149
Hsd3b4; Gm10681 ; Gm4450	-6.82	0.000	-2.47	0.025	-1.10	0.152	-1.38	0.077	1.19	0.149
Cyp2c53-ps	-7.19	0.000	-1.71	0.195	1.04	0.306	-1.17	0.383	1.04	0.992
Selenbp2	-7.35	0.007	-2.65	0.010	-1.19	0.721	-1.34	0.027	-1.15	0.096
Cyp2b10	-7.37	0.000	-8.03	0.001	1.55	0.005	2.01	0.002	-1.57	0.010
Cyp2c55	-8.96	0.001	-7.04	0.000	1.31	0.248	-1.44	0.244	-1.17	0.478
Cyp3a44	-9.04	0.000	-8.32	0.000	1.92	0.007	1.80	0.010	-1.91	0.002
Serpina4-ps1	-10.54	0.001	-5.72	0.010	-1.78	0.272	1.41	0.366	1.06	0.559
Gpr110	-14.78	0.000	-10.71	0.001	1.03	0.452	-1.14	0.155	1.08	0.597
Hsd3b5	-85.44	0.000	-3.80	0.026	1.62	0.462	-1.65	0.293	2.34	0.044

Supplemental Table S4. Cidea and Cidec expression over 9, 17, and 22 weeks

Linear fold changes in Cidea and Cidec genes shown with the longitudinal time periods of cohorts 1, 2, and 3 with their ANOVA P values.

Gene symbol	On week 9 Linear fold change (UN vs. NN)	ANOVA P value	On week 17 Linear fold change (UN vs. NN)	ANOVA P value	On week 22 Linear fold change (UN-Veh vs. NN-Veh)	ANOVA P value
Cidea	1.23	0.93	4.32	0.01	11.49	0.000
Cidec	-1.19	0.08	2.56	0.06	5.60	0.000

Supplemental Table S5. MBD sequencing at 22 weeks of age

Comparison of DNA methylation peaks among different groups; undernourishment *in utero* and normal nourishment *in utero* (cohort 3, UN-Veh vs. NN-Veh, n=4), undernourishment *in utero* with or without TUDCA (cohort 3, UN-TU vs. UN-Veh, n=4), and normal nourishment *in utero* with or without TUDCA (cohort 3, NN-TU vs. NN-Veh, n=4). Peaks detected include the overlapped promoter regions listed at the beginning (marked in blue) and the remainder of the genes. In UN-TU vs. UN-Veh, there was no significant methylation in the promoter region detected by MBD sequencing.

Gene symbol	Start	End	Fold changes (log2)	P value	Peak ID
**UN-Veh vs. NN-Veh**					
Fktn	53713923	53714857	-3.51	0.000	130874
Erdr1	90772977	90802259	1.33	0.000	208985
Mir466d	5378645	5379769	-1.29	0.000	102622
Scoc	83457938	83458821	0.87	0.000	185358
Tmem194	127663856	127665295	-0.64	0.000	24804
1700012D01 Rik	127663856	127665295	-0.64	0.000	24804
Csrnp2	100496429	100497267	-0.95	0.000	72575
Limd1	123461518	123462569	-2.08	0.000	202078
Erdr1	90802692	90808090	1.95	0.000	208986
Gphn	78350503	78351495	-1.68	0.000	41785
Mir6356	68691649	68692774	-1.11	0.000	94312
Fktn	53713923	53714857	-3.51	0.000	130874
Erdr1	90772977	90802259	1.33	0.000	208985
Filip1l	57390675	57392158	-1.75	0.000	77037
Mapk15	75997085	75999367	-1.00	0.000	68424
Lamp1	13153354	13153962	1.44	0.000	180149
Plekha1	130834036	130835035	-1.65	0.000	177567
Sun5	153857417	153857946	-1.57	0.000	112774
Hic2	17221798	17222962	-0.94	0.000	74009
Cacna1a	84491624	84492385	1.37	0.000	185501
Ins1	52324565	52325252	1.57	0.000	101126
Igsf9b	91096307	91097106	-1.24	0.000	78800
Myl12b	27323691	27324728	-0.72	0.000	192758
Cox10	70963256	70965204	-0.86	0.000	87181

Gene symbol	Start	End	Fold changes (log2)	P value	Peak ID
Itsn1	64071250	64072133	-0.83	0.000	29445
Ttll8	173724053	173725043	0.87	0.000	116272
Nus1	91764508	91765415	-0.99	0.000	78944
Rtn4rl1	88945800	88946557	0.91	0.000	70606
Prss39	52450644	52451736	-0.61	0.000	16889
Cltc	75263348	75267535	-0.27	0.000	30641
Mir3472	34499858	34500431	1.12	0.000	1533
Speer4d	49526773	49527401	-1.07	0.000	92408
Fmn2	86773101	86773860	1.29	0.000	31900
Myrf	145044789	145046357	-1.26	0.000	205497
P4ha2	118946071	118946734	1.09	0.000	150968
Mir6978	15606440	15607483	1.42	0.000	142099
Poc1a	174520606	174521211	1.21	0.000	11097
Vmn2r120	10268516	10270081	-0.55	0.000	97521
AI464131	54105997	54106660	1.41	0.000	28368
Amotl1	57213574	57214106	-1.08	0.000	85968
Cnp	106293344	106294510	0.75	0.000	199526
Fam163a	57587766	57588662	-1.53	0.000	86001
Arpc3	41505150	41506074	0.84	0.000	129644
Pdf	14638799	14639573	1.18	0.000	191856
Gm13498	100563210	100566194	-0.40	0.000	33574
Glb1l2	156199742	156200510	1.30	0.000	9687
Mdm2	122397373	122398768	0.79	0.000	151644
Tnfrsf19	107047828	107049043	-0.86	0.000	187437
Herc2	50913289	50914126	1.02	0.000	106022
Tmtc1	82770658	82773093	0.56	0.000	174165
Wwc2	26828587	26829231	1.16	0.000	192712
Panx1	117689249	117689782	1.13	0.000	23223
Scoc	61087221	61087866	1.33	0.000	59999
Zfp536	56099667	56100691	-0.58	0.000	172137
E2f7	148464874	148465508	1.36	0.000	167034
Erdr1	47972783	47973702	-1.02	0.000	183204
Vmn1r142	9063359	9063912	1.34	0.000	117416
Mydgf	15068103	15068714	-0.98	0.000	191916
Irgc1	83457938	83458821	0.87	0.000	185358
Hist1h4m;LOC102641229	37778331	37778777	1.28	0.000	170789
Foxk2	110651808	110652615	1.12	0.000	22490
Rab28	90808276	90809476	1.55	0.000	208987

Gene symbol	Start	End	Fold changes (log2)	P value	Peak ID
9530036O11 Rik	22151637	22153334	1.52	0.000	168642
Amy2a1	56186539	56191565	-0.27	0.000	85794
Csrnp2	108680192	108680649	1.09	0.000	134244
Olfr1298	24434537	24435083	1.19	0.000	168827
Kctd1	21812408	21820580	0.87	0.000	47234
Mpp7	96105776	96106927	0.86	0.000	71576
Dab1	121296510	121297360	-0.92	0.000	37034
Tmem30c	41342063	41342994	0.81	0.000	144024
Htt	28436496	28437466	-0.62	0.000	142919
Tns1	113568021	113568594	-1.25	0.000	124188
Pik3c3	100496429	100497267	-0.95	0.000	72575
Zbtb25	26388173	26389546	0.93	0.000	142865
Azi2	111649428	111650657	-0.62	0.000	109971
**UN-TU vs. UN-Veh**					
Cyp2b19	26776391	26777360	-1.16	0.000	169166
Rfk	17474718	17475180	1.38	0.000	97943
Endov	119507005	119507862	1.20	0.000	36723
Brca1	101521267	101521731	1.52	0.000	33747
Mir187	24426219	24427033	1.22	0.000	90825
Phf20	156253676	156254360	1.37	0.000	113058
Parvb	84248490	84249651	0.97	0.000	69875
4930401O10 Rik	71976540	71977735	0.97	0.000	30343
Oprm1	6630223	6631046	1.77	0.000	13842
Efl1	82693038	82693906	1.17	0.000	174149
Epg5	77932919	77933508	1.37	0.000	95435
Aebp2	140606177	140606873	1.45	0.000	165984
Tmem50a	134894341	134895234	0.98	0.000	137289
Erlin1	44076286	44077152	1.08	0.000	100279
Ntrk2	59130975	59131904	1.20	0.000	51280
St8sia1	143001453	143002324	-1.00	0.000	166223
Tex29	12057066	12057862	-1.01	0.000	179928
Adcy7	88265024	88265737	1.18	0.000	185901
**NN-TU vs. NN-Veh**					
Mir101c	2999847	3038796	-0.52	0.000	191357
Gm32511	71168994	71169960	1.12	0.000	196740
Pop5	115233364	115234316	1.08	0.000	150128
Ccl3	83649639	83650036	-1.17	0.000	31589
Ehmt1	24920489	24921215	1.06	0.000	103685

Gene symbol	Start	End	Fold changes (log2)	P value	Peak ID
Mettl24	40680593	40681422	1.06	0.000	16028
Mrpl12	120482602	120483553	0.86	0.000	36908
Col11a2	34038355	34039187	1.17	0.000	83676
Gpx4	80047171	80048215	0.88	0.000	19810
Mir6408	60921682	60922428	1.03	0.000	17536
Lonp2	86609518	86610264	1.42	0.000	185716
Dgki	37270029	37270769	1.40	0.000	158343
Ptk7	46618627	46620070	0.85	0.000	84834
Mir1967	124043846	124044484	2.10	0.000	190670
Smim20	53283494	53284109	1.59	0.000	144581
Tmem200a	26126677	26127471	-1.29	0.000	15424
Mir6956	78987169	78988916	0.70	0.000	68954
Fam209	172490372	172491354	1.26	0.000	115962
Mir763	120427044	120427733	1.43	0.000	23808
Mir1249	84942340	84943269	1.37	0.000	70043
Saa4	46730472	46731548	1.14	0.000	171671
Has3	106806729	106807291	1.47	0.000	187404
Rgs9	109271003	109271997	1.15	0.000	34861
Zfp827	79050577	79051269	1.36	0.000	185073
Clint1	45893554	45894537	1.20	0.000	27563
Pex5l	33121242	33121769	1.48	0.000	118777
Aqp9	71168994	71169960	1.12	0.000	196740
Cbr4	61624787	61625524	1.50	0.000	183939
Gm16675	46716300	46717031	1.32	0.000	182948
Ice1	70647742	70648635	1.21	0.000	52221
Cog2	124534967	124535890	1.15	0.000	190781
Nat3	67540556	67540992	-1.30	0.000	184175
Tmem132d	128263334	128264130	1.31	0.000	152556
Kdr	75980708	75981433	1.32	0.000	146235
Ppp2r1b	50827497	50828392	1.12	0.000	194738
1700011H14 Rik	49215133	49215794	-1.36	0.000	58972
Nfyb	82739941	82740638	1.36	0.000	20211
Rassf4	116690563	116691483	-2.97	0.000	163555
Lig3	82812190	82813510	0.96	0.000	31503
Lgi1	38251809	38253339	-0.48	0.000	99551
Zcchc14	121639125	121640176	0.93	0.000	190044
Paxip1	27789197	27790082	1.01	0.000	142898
Golga5	102471863	102472754	-0.82	0.000	43882
Grm3	9661632	9662356	1.45	0.000	141855

Gene symbol	Start	End	Fold changes (log2)	P value	Peak ID
E2f8	48886514	48887458	0.97	0.000	171802
Kl	150954865	150956220	0.87	0.000	156286
Pnliprp2	58771516	58772483	-0.93	0.000	102214
Vdr	97922625	97923149	1.48	0.000	72053
Dock4	40648269	40649080	-1.47	0.000	39484
Urad	147334650	147335220	1.43	0.000	155570
Elk3	93320808	93321551	1.26	0.000	21334
Pglyrp2	32415476	32417523	-0.50	0.000	83528
Neurl2	164836228	164840868	0.26	0.000	114170
Filip1l	57468236	57469266	-0.83	0.000	77052
Gpm6a	55371958	55375280	2.64	0.000	183591
4930579H20 Rik	27254379	27255662	-1.07	0.000	15484
Stpg2	139254565	139255511	-0.80	0.000	126188
Atf1	100169937	100170705	1.00	0.000	72497
Ankrd55	112281560	112282413	1.29	0.000	55226
Muc1	89231493	89232546	0.97	0.000	122243
Nxpe2	47955807	47956426	-1.26	0.000	194429
Csf2ra	61232582	61233568	-0.68	0.000	102362
Speer4d	15606440	15607483	1.51	0.000	142099
Gng4	13753224	13754188	-2.41	0.000	46867
1700112J05 Rik	77038245	77039191	0.99	0.000	146401
Cdh26	178468052	178469058	1.15	0.000	116610
Inpp4b	81406429	81407379	-1.02	0.000	185261
Gm20594	79657458	79658820	-0.92	0.000	160736
Ccm2l	153083411	153084012	1.32	0.000	112675
Rims2	39244399	39245696	1.66	0.000	66576
Tbxas1	38774301	38775198	-1.03	0.000	158513
Cdh15	122858948	122859824	0.99	0.000	190376
Nfkbiz	55813667	55814728	0.83	0.000	76903
Rusc1	89087685	89088529	1.37	0.000	122218
Glo1	30603496	30604566	0.81	0.000	83166
Slc39a8	135963426	135963995	-0.97	0.000	125898
Lyrm9	78852359	78852915	1.13	0.000	31102
4930432M1 7Rik	121672731	121673505	1.10	0.000	124651
5930412G12 Rik	128544030	128544732	1.11	0.000	152598
Edn1	42322847	42323587	1.09	0.000	48649

Gene symbol	Start	End	Fold changes (log2)	P value	Peak ID
Zfp365	67924945	67925983	-0.75	0.000	18319
Polr3c	96775398	96776162	1.05	0.000	122900
Sim2	94056372	94057324	0.96	0.000	79453
Gm4246; Tmem14c	41019014	41020539	0.78	0.000	48493
Sepp1	3169128	3170602	1.46	0.000	64177
Krr1	111931934	111932510	1.11	0.000	22669
Ajap1	153425976	153426968	0.92	0.000	140778
Unc5d	29559378	29560028	1.26	0.000	181843
Olfr92	37105458	37106417	-0.66	0.000	84046
Itpkc	27216753	27217566	1.01	0.000	169210
Ebf3	137230719	137231586	-0.67	0.000	178313
Picalm	90167394	90168160	1.01	0.000	174448
Scaf4	90312435	90313347	0.98	0.000	78606
A930005H10Rik	115895910	115896434	1.13	0.000	124260
Ces5a	93525203	93525800	-1.00	0.000	186468
Mest	30726879	30727925	2.55	0.000	157824
Dhx37	125415889	125416875	0.81	0.000	152297
Slc35d1	103226101	103227316	0.79	0.000	133641
E2f7	110741848	110742682	1.03	0.000	22503
Secisbp2l	125760518	125761345	1.06	0.000	110931
Mark2	7352650	7353545	1.06	0.000	97273
Srgap1	121968457	121969213	1.03	0.000	24156
Sipa1l1	82280692	82281622	-0.87	0.000	42169
Twsg1	65957908	65958395	1.11	0.000	86557
Lypd6	50004667	50005472	-1.06	0.000	105980
Lrrc47	154006800	154007827	0.79	0.000	140921
Scd2	44271969	44272721	1.22	0.000	100310
Adrb2	62118059	62119018	0.99	0.000	93511
Twist2	91752715	91753984	0.66	0.000	5901
Mfsd9	40784103	40785048	0.90	0.000	2424
Gbp9	105112996	105114090	-0.58	0.000	148553
Cog7	121958959	121959417	1.34	0.000	176556
Fbln7	128853038	128853544	1.18	0.000	111244
Arl15	113919891	113920794	0.88	0.000	55500
Kcnk2	189492141	189493136	-0.67	0.000	12940
Spata18	73703668	73704650	0.76	0.000	145902
Ank3	69693812	69694533	1.02	0.000	18562
Psmg4	34165380	34167711	0.65	0.000	47985

Gene symbol	Start	End	Fold changes (log2)	P value	Peak ID
Cand1	119191369	119192107	1.28	0.000	23525

Supplemental Table S6. Summary of 7 GOIs and their biological relevance.

Biological relevance of 7 GOIs was reviewed and briefly discussed below with corresponding citations.

Gene Symbol	Biological Function
Rgs16	Regulator of G Protein Signaling has been reported to be related with the onset of diabetes and play a role in hepatic fatty acid oxidation[1].
Themis	Thymocyte Selection Associated gene is reported to plays a central role in late thymocyte development by controlling both positive and negative T-cell selection[2].
Ifi27l2b	Interferon alpha-inducible protein 27-like protein 2B is involved in apoptotic pathways[3].
Cyp17a1	Cytochrome P450 Family 17 Subfamily A Member 1 is reported to be actively involved in pathways like oxidative stress which is likely to play a keen role in development of hepatic steatosis[4].
Sprr1a	Small Proline Rich Protein 1A, a Protein Coding gene. Among its related pathways are developmental biology and keratinization involved in structural molecule activity and protein binding, bridging[5].
Ly6d	Lymphocyte antigen 6D is a B-cell marker at early stage of development[6].
Orm3	Orsomucoid subunit 3 is known as immune-modulator was reported to be expressed in adipocytes. Orm-3 was reported to integrate inflammatory and metabolic signals to preserve energy homeostasis[7].

1 Pashkov, V. *et al*. Regulator of G protein signaling (RGS16) inhibits hepatic fatty acid oxidation in a carbohydrate response element-binding protein (ChREBP)-dependent manner. *J Biol Chem* 286, 15116-15125 (2011).

2 Kakugawa, K. *et al*. A novel gene essential for the development of single positive thymocytes. *Mol Cell Biol* 29, 5128-5135 (2009).

3 Lu, M. Y. & Liao, F. Interferon-stimulated gene ISG12b2 is localized to the inner mitochondrial membrane and mediates virus-induced cell death. *Cell Death Differ* 18, 925-

936 (2011).

4 Liu, Y. *et al.* The role of cytochrome P450 in nonalcoholic fatty liver induced by high-fat diet: a gene expression profile analysis. *Zhonghua Gan Zang Bing Za Zhi* 25, 285-290 (2017).

5 Gibbs S. *et al*. Molecular characterization and evolution of the SPRR family of keratinocyte differentiation markers encoding small proline-rich proteins. *Genomics* 16 (3), 630-7 (1993).

6 Inlay, M. A. *et al*. Ly6d marks the earliest stage of B-cell specification and identifies the branchpoint between B-cell and T-cell development. *Genes Dev* 23, 2376-2381 (2009).

7 Lee, Y. S. *et al.* Adipocytokine orosomucoid integrates inflammatory and metabolic signals to preserve energy homeostasis by resolving immoderate inflammation. *J Biol Chem* 285, 22174-22185 (2010).

Supplemental Table S7. List of antibodies
List of specific antibodies used in immunohistochemistry and real-time quantitative PCR for Chromatin Immuno Precipitation (ChIP) assays in the vicinity of the Cidea and Cidec genes with supplier names.

Name of the antibody	Supplier	Cat no.	Clone no.
**For immunohistochemistry**			
Cide-a antibody	Novus Biologicals, USA	NBP1-76950	Polyclonal
Cide-c antibody	Bioss Antibodies Inc., Massachusetts, USA	Bs-6796R	Polyclonal
**For ChIP assays**			
Anti-histone H3 (di methyl K4) antibody [Y47] - ChIP grade	Abcam, Cambridge, MA, USA	ab 32356	Monoclonal
Anti-monomethyl-Histone H3 (Lys9) Antibody	Merck, Germany	#07-450	Polyclonal
Anti-dimethyl-histone H3 (Lys9) antibody	Merck, Germany	#07-441	Polyclonal
Anti-tri-methyl-Histone H3 (Lys9) antibody	Merck, Germany.	#07-442	Polyclonal
Anti-histone H3 (tri methyl K9) antibody - ChIP Grade	Abcam, Cambridge, MA, USA	ab8898	Polyclonal
Anti-acetyl H3 (Lys9) antibody	Merck, Germany	#07-352	Polyclonal
Anti-histone H3 (mono methyl K36) antibody - ChIP Grade	Abcam, Cambridge, MA, USA	ab 9048	Polyclonal
Anti-histone H3 (di-methyl K36) antibody-ChIP grade	Abcam, Cambridge, MA, USA	ab 9049	Polyclonal
Anti-monomethyl-Histone H3 (Lys27) antibody	Merck, Germany	#07-448	Polyclonal
Anti-histone H3 (di-methyl K27) antibody-ChIP grade	Abcam, Cambridge, MA, USA	ab 24684	Polyclonal

Name of the antibody	Supplier	Cat no.	Clone no.
Anti-tri-methyl-histone H3 (Lys27) antibody	Merck, Germany	#07-449	Polyclonal
Anti-histone H3 (tri-methyl K27) antibody - ChIP Grade	Abcam, Cambridge, MA, USA	ab6002	Monoclonal
Anti-acetyl-histone H4 antibody	Merck, Germany	#06-598	Polyclonal
Anti-histone H4 (tri-methyl K20) antibody - ChIP Grade	Abcam, Cambridge, MA, USA	ab9053	Polyclonal
Normal Rabbit IgG as a control	CST (Cell Signaling Technology), Japan	#2729	Polyclonal

Supplemental Table S8. List of primers
List of oligonucleotide primers used in real-time quantitative PCR (qPCR) and Chromatin Immuno Precipitation (ChIP) assays in the vicinity of the Cidea and Cidec genes in mouse liver tissue.

Name	Sequence	Supplier
Cidea primer (approximate region) -2000 forward	ACTTTCTCCCTCCTCTTCCA	Fasmac Co., Ltd., Japan
Cidea primer (approximate region) -2000 reverse	GTCATGCAGACTTGAAAGCA	Fasmac Co., Ltd., Japan
Cidea primer (approximate region) -1000 forward	CATTATTCCAGCCCTGAGACA	Fasmac Co., Ltd., Japan
Cidea primer (approximate region) -1000 reverse	CCTCCAAGCTCACAGATAGAGA	Fasmac Co., Ltd., Japan
Cidea primer (approximate region) -500 forward	CACTGGGAAACTGCTTTTAGC	Fasmac Co., Ltd., Japan
Cidea primer (approximate region) -500 reverse	GGAGTTGCTAGTTTTCTGCTCA	Fasmac Co., Ltd., Japan
Cidea primer (approximate region) +1 forward	GCTTCTCTACTGCCAAGCCATA	Fasmac Co., Ltd., Japan
Cidea primer (approximate region) +1 reverse	CAGGATTTGGTGATGTTGGTG	Fasmac Co., Ltd., Japan
Cidea primer (approximate region) +1000 forward	GCTAAGGCAAGAGACTTGGAGA	Fasmac Co., Ltd., Japan
Cidea primer (approximate region) +1000 reverse	TATAGGGAGGCCCTGTATGTCT	Fasmac Co., Ltd., Japan
Cidea primer (approximate region) +2000 forward	ATGGGAACATCCTTGCGTCT	Fasmac Co., Ltd., Japan
Cidea primer (approximate region) +2000 reverse	CAAGGGAGGGAGTCATAGATCA	Fasmac Co., Ltd., Japan
Cidea primer (approximate region) +5000 forward	ACCTGTGTACCACCTCAGGATA	Fasmac Co., Ltd., Japan
Cidea primer (approximate region) +5000 reverse	CCATATAGCACAGCAGGACAGA	Fasmac Co., Ltd., Japan
Cidea primer (approximate region) Over +5000 forward	AGTTTCCTTCAACCTGGGAAC	Fasmac Co., Ltd., Japan
Cidea primer (approximate region) Over +5000 reverse	GCAGAGTTTTCCAGGAATGG	Fasmac Co., Ltd., Japan
Cidec primer (approximate region) -2000 forward	ACTCAATGGGTCATCTTGCTG	Fasmac Co., Ltd., Japan
Cidec primer (approximate region) -2000 reverse	CTAATTCAAGACAAGCCTGAGC	Fasmac Co., Ltd., Japan

Name	Sequence	Supplier
Cidec primer (approximate region) -1000 forward	GAACTCATTCCCCATGCTCTT	Fasmac Co., Ltd., Japan
Cidec primer (approximate region) -1000 reverse	CCAGGCTTCCCTCCATTTTAT	Fasmac Co., Ltd., Japan
Cidec primer (approximate region) -500 forward	ATGACACAAGCTGTACCACAGG	Fasmac Co., Ltd., Japan
Cidec primer (approximate region) -500 reverse	GCTCACATGCCTGTTTTCCT	Fasmac Co., Ltd., Japan
Cidec primer (approximate region) +1 forward	CCTGAGACAGGGACAAATCAGT	Fasmac Co., Ltd., Japan
Cidec primer (approximate region) +1 reverse	GCCTCACTCACTCCATTGTTT	Fasmac Co., Ltd., Japan
Cidec primer (approximate region) +1000 forward	CACCATTGTGGTTCCTGTCAT	Fasmac Co., Ltd., Japan
Cidec primer (approximate region) +1000 reverse	CTTGCCAGTTTCCCCATCTAT	Fasmac Co., Ltd., Japan
Cidec primer (approximate region) +2000 forward	ACAGAGAAACCCTGTCTCGAA	Fasmac Co., Ltd., Japan
Cidec primer (approximate region) +2000 reverse	TCACTTGACCATGTCACCTCTC	Fasmac Co., Ltd., Japan
Cidec primer (approximate region) +5000 forward	CCTCATCCCACAAATAAACACA	Fasmac Co., Ltd., Japan
Cidec primer (approximate region) +5000 reverse	ACCACCCTATTCCAACAATCC	Fasmac Co., Ltd., Japan
Cidec primer (approximate region) Over +5000 forward	TTCTGAGTTCGAAGCCAGTCT	Fasmac Co., Ltd., Japan
Cidec primer (approximate region) Over +5000 reverse	TTTCGAGACAGGGTTTCTCTG	Fasmac Co., Ltd., Japan
